# Clock model makes a large difference to age estimates of long-stemmed clades with no internal calibration: a test using Australian grasstrees

**DOI:** 10.1186/s12862-014-0263-3

**Published:** 2014-12-19

**Authors:** Michael D Crisp, Nate B Hardy, Lyn G Cook

**Affiliations:** Research School of Biology, The Australian National University, Acton, Canberra, ACT 2601 Australia; Department of Entomology and Plant Pathology, Auburn University, Auburn, AL 36849 USA; The University of Queensland, School of Biological Sciences, Brisbane Qld, 4072 Australia

**Keywords:** Divergence times, Substitution rate heterogeneity, Uncorrelated lognormal clock, Random local clocks, Relative rates, Life history, *Xanthorrhoea*

## Abstract

**Background:**

Estimating divergence times in phylogenies using a molecular clock depends on accurate modeling of nucleotide substitution rates in DNA sequences. Rate heterogeneity among lineages is likely to affect estimates, especially in lineages with long stems and short crowns (“broom” clades) and no internal calibration. We evaluate the performance of the random local clocks model (RLC) and the more routinely employed uncorrelated lognormal relaxed clock model (UCLN) in situations in which a significant rate shift occurs on the stem branch of a broom clade. We compare the results of simulations to empirical results from analyses of a real rate-heterogeneous taxon – Australian grass trees (*Xanthorrhoea*) – whose substitution rate is slower than in its sister groups, as determined by relative rate tests.

**Results:**

In the simulated datasets, the RLC model performed much better than UCLN: RLC correctly estimated the age of the crown node of slow-rate broom clades, whereas UCLN estimates were consistently too young. Similarly, in the *Xanthorrhoea* dataset, UCLN returned significantly younger crown ages than RLC (mean estimates respectively 3–6 Ma versus 25–35 Ma). In both real and simulated datasets, Bayes Factor tests strongly favored the RLC model over the UCLN model.

**Conclusions:**

The choice of an unsuitable molecular clock model can strongly bias divergence time estimates. In particular, for data predicted to have more rate variation among than within clades, dating with RLC is much more likely to be accurate than with UCLN. The choice of clocks should be informed by the biology of the study group (e.g., life-form) or assessed with relative rate tests and post-hoc model comparisons.

**Electronic supplementary material:**

The online version of this article (doi:10.1186/s12862-014-0263-3) contains supplementary material, which is available to authorized users.

## Background

Molecular-clock dating of long-stemmed clades (“broom” or “stemmy” clades [1]) is prone to large estimation errors partly because calibration points can be placed only at nodes, not part-way along stems [2,3]. Likewise, shifts in substitution rate along lineages can be detected only at nodes, and cannot be placed within unbroken stems. As molecular dating depends upon accuracy of rate modelling, inferred rate variation within long branches has been hypothesized to contribute to dating error, e.g. in the crown node of angiosperms [4]. Calibration is critically important for reducing error in molecular dating [5-7], for instance by improving estimates of rate heterogeneity among lineages [8]. Conversely, estimation error is greater in target clades when calibrations are experimentally omitted [9]. Furthermore, abrupt rate shifts in some lineages of a phylogeny, followed by rate conservation, can mislead rate and age estimation, even when the node of interest is calibrated [9].

A strict molecular clock has been rejected in analyses of most empirical datasets, and is inapplicable to data in which rate shifts occur, so relaxed molecular clock models are used widely (reviewed in [10]). Commonly used relaxed clocks include uncorrelated, autocorrelated and local clocks. In an autocorrelated clock, the rate along a given branch is more similar to its parent branch than a branch chosen at random, though autocorrelation models differ in the degree to which they restrict rate variation between parent and daughter branches. A local clocks model differs from an autocorrelated clock by setting a single rate throughout a contiguous section of the tree, allowing relatively few such clocks in different parts of the tree, with abrupt shifts in rate between these clocks. An uncorrelated clock has a distinct rate along each branch, with the rates drawn at random from a specified probability distribution, thus allowing random and frequent rate shifts. The uncorrelated lognormal (UCLN) model draws its rates from a lognormal distribution and appears to be more robust to violation of assumptions about clock rate variation, and a better fit to simulated and empirical datasets, than are strict or autocorrelated clock models [11,12]. Moreover, studies differ in whether they find support for uncorrelated clocks [11,12] or correlated clocks [13]. If rates shift infrequently and are conserved along lineages, then dating using either an uncorrelated or correlated model could be inaccurate and local models can be a better fit [9,10,14]. Having the means to detect if and when rates shift, and to correct branch lengths accordingly, could be critically important to evolutionary analysis using molecular phylogenies.

The random local clocks (RLC) model proposes and compares a series of alternative local molecular clocks, each potentially arising on any branch and extending over a contiguous part of the phylogeny. In BEAST, the RLC model parameters (rates and shift points) are estimated simultaneously with phylogenetic relationships in a Bayesian framework [10,15]. In a real and a simulated phylogeny that included abrupt and sustained rate shifts, RLC outperformed UCLN in terms of accuracy of divergence-time estimates, and UCLN estimates were misleadingly precise [9].

To date, there have not been comparisons of RLC and UCLN models based on multiple phylogenies exhibiting rate heterogeneity among lineages. As modelling of DNA substitution rates directly affects estimation of dates, the assumptions of underlying rate models should be biologically realistic. For example, if substitution rates depend upon certain life history traits, then abrupt shifts in these traits might suggest an RLC clock model is preferable to UCLN. Although the determinants of rate variation among lineages are often unclear [16-18], undoubtedly there are lineage-specific effects that result from shifts in life history and life forms in different clades of organisms. Among mammals, generation time is strongly negatively correlated with substitution rate, and is explained by the sequestering of the female germ line (egg cells) early in the development of the female [17,19]. Plants also show a negative relationship between substitution rate and generation time [16,18,20], though the primary cause is unclear because, in contrast to mammals, female gametes are produced multiple times during the life of the individual. Recently, it has been shown that plant height correlates better with rate than does generation time – taller plants have slower rates [20]. These authors suggest that the rate of mitosis in apical meristems may be the determining factor because taller plants have slower growth at their tips.

Here, we use a simulation approach to evaluate the performance of RLC and UCLN models. We simulate multiple trees having a long-stemmed clade with a known crown age, and then simulate DNA datasets on these trees so that there is a large downward shift in substitution rate associated with the long stem. We compare the accuracy of the RLC and UCLN clock models in retrieving the known crown age of the long-stemmed clade, and also use marginal likelihoods to determine which model is the better fit. We compare these results with those we obtain using Australian grass trees (*Xanthorrhoea*), a group that is expected to show a slower substitution rate than their relatives. As the crown age of *Xanthorrhoea* is unknown, we compare the performance of the UCLN and RLC clock models using marginal likelihoods to determine which is the better fit. *Xanthorrhoea* is a genus of arborescent grasstrees and a characteristic plant of the fire-dependent sclerophyll biome with nutrient-poor soils in western, southern and eastern Australia, sometimes dominating the shrub layer. It is the only genus in Xanthorrhoeaceae subfamily Xanthorrhoeoideae. Extant *Xanthorrhoea* appears to have undergone major morphological and ecological transformations compared with the last common ancestor that it shared with its worldwide sister groups (Xanthorrhoeaceae subfamilies Hemerocallidoideae and Asphodeloideae). The latter clades have diverse morphologies and life histories but most are smaller and faster-maturing plants than *Xanthorrhoea*.

Several traits in *Xanthorrhoea* lead us to predict that this lineage evolves more slowly than its sister groups. The plants have a palm-like form with one or few apical meristems and are long-lived (up to 450 yr) with slow apical growth (10–20 mm p.a.) [21] and reproductive maturity may not be reached until 20–30 years of age [22]. Reproduction in *Xanthorrhoea* occurs mainly in the first spring following a wildfire [21,23] (Additional file 1: Figure S1) and, in the sclerophyll biome, estimates of intervals between fires range from 3–5 to 10–25 yr [21,22,24]. Thus, generation time is likely longer even than is indicated by the slow growth rates alone. It has been suggested that both generation time (which is linked to longevity) and the rate of apical growth (which is linked with the rate of mitosis) are negatively related to DNA substitution rate in plants [20]. Mutations occur when DNA is replicated and cells divide (by meiosis and mitosis) and are passed to the next generation; thus variation in both generation time [17] and plant height [20] are likely to explain variation in DNA substitution rates. We test the hypothesis of a substitution rate shift downwards in *Xanthorrhoea* by using the well-known Relative Rates test that assumes no phylogeny [25], followed by a phylogenetic test that corrects for the confounding effect of within-clade rate variation (Local Clock Permutation Test [26]). We then use Bayesian phylogenetics (BEAST) to estimate the crown age for *Xanthorrhoea* with the same combinations of clock and tree models as used on the simulated datasets. We consider the implications of our results for the estimation of clade ages when rates vary among lineages.

## Results

### Simulations of strong among-clade substitution rate variation

**Figure 1:**
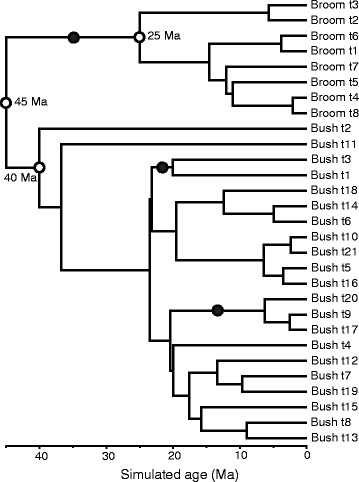
**Example of a simulated chronogram (Tree 1 of 10).** Tree parameters were based on those of Xanthorrhoeaceae and were simulated using TESS using a birth-death model, and “broom” and “bush” sister clades. The age of the root was fixed at 45 Ma, that of the broom crown node at 25 Ma, as marked with open circles. DNA sequences were then simulated along the branches of the trees using a GTR + Γ substitution model with slow (broom clade) and fast (bush clade) rates (Figure 2), as in *Xanthorrhoeaceae*, to test the relative performance of the UCLN and RLC clock models. The simulated trees varied in size from 19 to 42 terminals, and also in the proportion of terminals in the bush vs broom clades, from 31 vs 4 to 6 vs 20. For the BEAST analyses, three log-normal age-calibration priors were used: one was placed on the stem of the broom clade and two were placed on the stem of each of two clades internal to the bush clade (marked with closed circles). The mean and offset of each calibration prior were equal to half the distance between the crown and stem nodes of the respective clade.

**Figure 2:**
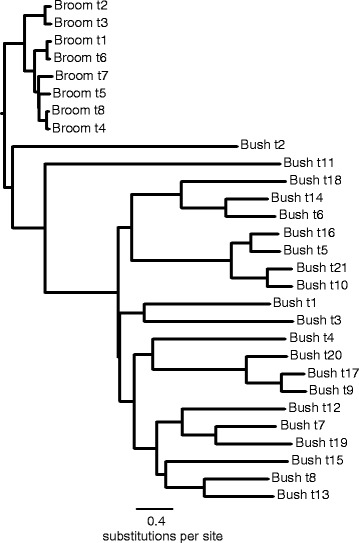
**A maximum likelihood phylogram estimated from DNA sequences simulated on the tree in Figure** **1** **.** We simulated a 1 Kb sequence set using the R package PhyloSim with a GTR + Γ model of substitution (as for Xanthorrhoeaceae), with the alpha parameter set to 1. Substitution rates were modelled by setting the probability of each particular substitution in a Q matrix, with a probability 10 times higher in the bush clade than in the broom clade. See text for the full set of parameter values. Branch lengths are proportional to the substitution rates and illustrate the overall 10x rate difference between the broom and bush clades, and additionally, the stochasticity built into the model. The phylogram was generated in CIPRES using RAxML with a GTR + Γ model.

**Figure 3:**
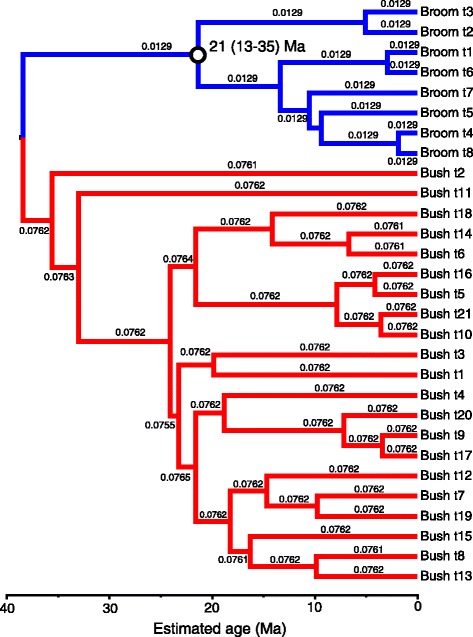
**Reconstruction of the simulated chronogram (Figure** 1 **) using BEAST with the RLC clock model and calibration priors as shown on Figure** **1** **.** Each estimate assumed a GTR + Γ substitution model. Branches are colored by inferred local clock rates in the introns partition: red = fast, blue = slow, and each branch is labelled with the estimated rate (substitutions site^−1^ Myr^−1^). Estimated age with 95% BCI is shown for the broom crown node (open circle).

**Figure 4:**
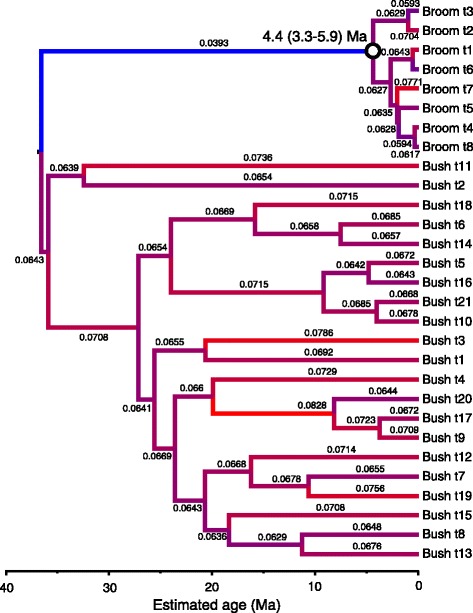
**Reconstruction of the simulated chronogram (Figure** 1 **) using BEAST with the UCLN clock model and calibration priors as shown on Figure** **1** **.** Each estimate assumed a GTR + Γ substitution model. Branches are colored by inferred local clock rates in the introns partition: red = fast, blue = slow, and each branch is labelled with the estimated rate (substitutions site^−1^ Myr^−1^). Estimated age (with 95% BCI) is shown for the broom crown node (open circle).

**Figure 5:**
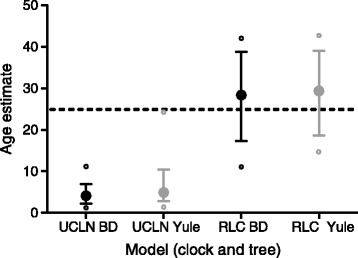
**Estimated crown ages of long-stemmed “broom” clades in simulated trees using BEAST with UCLN and RLC clock models in combination with Birth-death (BD, black) and Yule (grey) phylogenetic tree models.** Trees were simulated using TESS with a BD model and sequences simulated on the trees using PhyloSim with a local clock model in which the substitution rate was 10x slower in the broom clade than in its sister “bush” clade. Each large dot represents the mean age across 10 simulated trees, error bars are mean 95% BCIs, and small open circles represent the extreme 95% BCIs across all 10 datasets. The horizontal broken line shows the true age (25 Ma) of the broom crown node.

Our simulated datasets were designed to mirror our understanding of the evolution of the Xanthorrhoeaceae, i.e. a phylogenetic tree including a long-stemmed “broom” clade, a slow molecular clock and a crown with no internal calibration, and a “bush” sister clade with a short stem, faster clock and internal calibration (e.g., Figures 1–2). The major difference was that, in the simulated dataset, the crown age of the slow-clock clade was known in advance. As expected, in the BEAST analyses, the RLC clock model (Figure 3) outperformed UCLN (Figure 4) and accurately reconstructed the abrupt and sustained change in substitution rate occurring along the long internal branch of the broom clade (at least under the simulation conditions used here). The true crown age of the broom clade in each simulated dataset was 25 Ma. Under RLC, the mean age estimate for this node was 28.4 Ma (95% BCI = 11–42 Ma) with a Birth-death phylogenetic tree model, and 29.4 (14–43) Ma with a Yule model (Figure 5, Additional file 2: Table S1). In all RLC analyses, the true age fell within the 95% BCI of the estimate. By contrast, under UCLN, the mean estimates were much younger, being 4.1 (0.5-11) Ma (Birth-death) and 4.9 (0.5-10) Ma (Yule), and the upper (older) limit of the 95% BCI never contained the true age (Figure 5, Additional file 2: Table S1). Age estimates under the two clock models were significantly different (Mann–Whitney test, *P* < 0.0001; H_0_ = difference in means is 0) for both tree models (Birth-death and Yule). By contrast, comparisons between the Yule and Birth-death age estimates within each clock model did not find significant differences (Mann–Whitney test, *P* = 0.912 for RLC and P = 0.0524 for UCLN; see also Figure 5). Under RLC (with both tree models), the number of rate shifts inferred was small (median = 1, mean = 1.2-1.5) across all ten simulated datasets. The simulated trees varied in size from 19 to 42 terminals, and in the proportion of terminals in the bush vs broom clades, from 31 vs 4 to 6 vs 20 (Additional file 2: Table S1). There was no evidence that the date estimates showed a systematic relationship with either tree size (number of terminals) or relative clade sizes. For all 10 simulated datasets, Bayes factors calculated from marginal likelihoods found “very strong” support (BF > 10 [27]) for RLC over UCLN under both the Birth-death and Yule phylogenetic tree models.

### Xanthorrhoeaceae phylogeny

**Figure 6:**
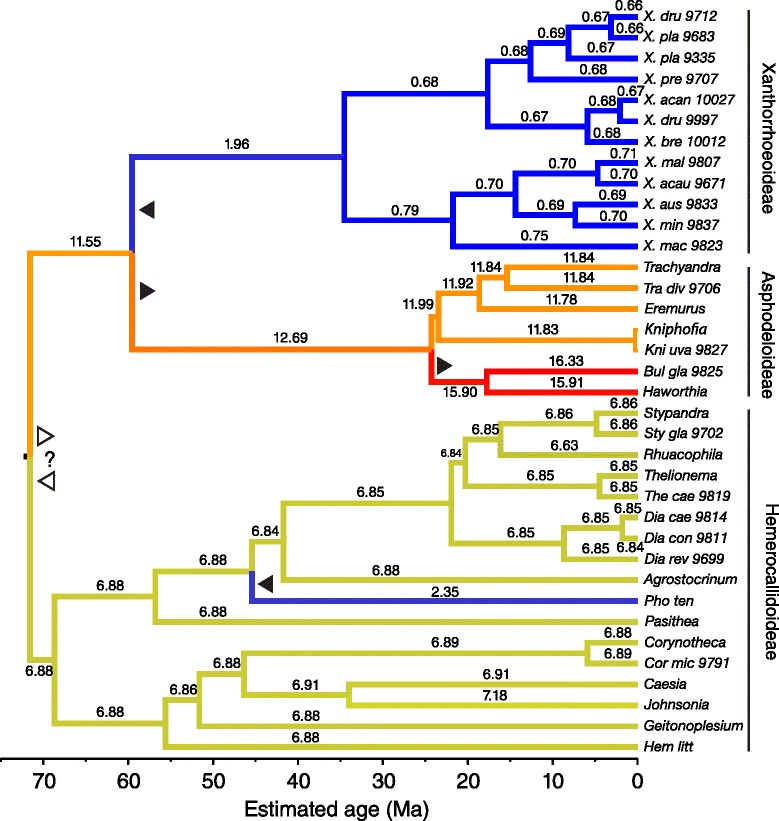
**Random local clocks (RLC) chronogram of Xanthorrhoeaceae from combined** ***ndhF*** **and** ***trnL*** **-** ***trnF*** **(chloroplast DNA) sequences.** Analysis used BEAST with a Yule tree model. Branches are colored by inferred local clock rates: red = fastest, blue = slowest, orange and green = intermediate, and each branch is labelled with the actual rate (substitutions site^−1^ Myr^−1^ × 10^4^). Filled pointers show likely rate shifts (to right = upwards, to left = downwards); open pointers show possible alternatives. Clade labels with bars indicate subfamilies. The scale indicates time before present (Ma).

**Figure 7:**
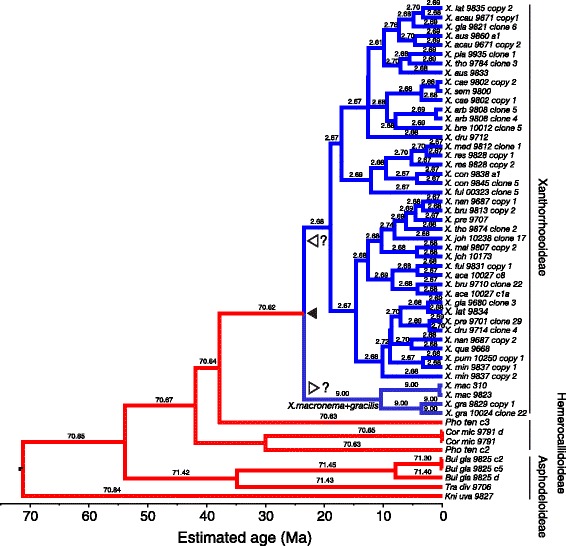
**RLC chronogram of Xanthorrhoeaceae derived from sequences of** ***rpb2*** **(nuclear DNA).** Analysis used BEAST with a Yule tree model. Branches are colored by inferred local clock rates in the introns partition: red = fast, blue = slow, and each branch is labelled with the actual rate (substitutions site^−1^ Myr^−1^ × 10^4^). Pointers show the two inferred rate shifts: one downwards in the MRCA of Xanthorrhoeoideae (filled pointer), followed by a second shift (open pointers), which was either a further downward shift in the stem of the large upper clade or a small upward shift in the stem of the *X. macronema + X. gracilis* clade. The pattern of exon rates and shifts is essentially similar but slower (Additional file 3: Figure S11). Clade labels with bars indicate subfamilies. The scale indicates time before present (Ma).

To derive a secondary age calibration for the crown node of Xanthorrhoeaceae, a phylogenetic dating analysis was carried out on a broad sample of monocots including multiple fossil calibration points and using sequences of the chloroplast regions *ndhF* and *trnL-F*. The resulting topologies of both the RAxML and BEAST trees (Additional file 3: Figures S2 and S3) were congruent with published trees [28-30] and returned strong support for monophyly of Xanthorrhoeaceae and its subfamilies, and for a sister group relationship between *Xanthorrhoea* and Hemerocallidoideae. Partitioned and non-partitioned models made little difference to the topologies, except in clades with poor resolution, e.g., within the crown of *Xanthorrhoea*. The maximum likelihood (RAxML) analysis indicated rate heterogeneity within Xanthorrhoeaceae, insofar as Xanthorrhoeoideae have much shorter branches than in the other two subfamilies (Additional file 3: Figure S2).From the Xanthorrhoeaceae-only datasets, which were comprehensively sampled at the species level, cpDNA and *rpb2* both yielded maximum likelihood trees in which the branch lengths were conspicuously shorter within *Xanthorrhoea* than in the sister groups (Additional file 3: Figures S4 and S5). The cpDNA tree differs from the monocot tree in placing *Xanthorrhoea* sister to Asphodeloideae but with weak support (bootstrap = 67). In contrast, the *rpb2* tree shows *Xanthorrhoea* nested within successively paraphyletic groups of Hemerocallidoideae and Asphodeloideae. Paralogous copies of *rpb2* are evident, e.g. clones from individuals of *Phormium* are in separate lineages (Additional file 3: Figure S4), though *Xanthorrhoea* is monophyletic (bootstrap = 100). With both loci and under both clock models, BEAST gave topologies (Figures 6 and 7, Additional file 3: Figures S6-S10) that were substantively the same as those from RAxML (Additional file 3: Figures S4 and S5), at least for well-supported nodes.

### Bayesian model fitting in Xanthorrhoeaceae

*A posteriori* Bayes factor tests using the marginal likelihoods from the BEAST analyses found a clear preference among alternative models in all three datasets (Table 1). Partitioned loci were preferred to non-partitioned “very strongly” [27] in both the monocots-cpDNA and Xanthorrhoeaceae-*rpb2* datasets, while non-partitioned loci were preferred in the Xanthorrhoeaceae-cpDNA dataset. All tests indicated a preference for Yule over Birth-death tree models, very strongly in most cases. Comparison of clock models in the Xanthorrhoeaceae-only datasets (cpDNA and *rpb2*) found a strong preference for RLC over UCLN in both loci (Table 1). Clock comparisons could not be made in the large monocot-cpDNA dataset because twelve separate analyses using RLC did not reach stationarity or convergence after 300 M generations. This failure is not surprising given reports of similar difficulties with estimating the parameters for this model [9,15]. The problem appears to arise from inefficient sampling by the Markov chains when transitions between alternative states require large moves [9] and is exacerbated in large and/or complex datasets. Results presented from the BEAST analyses are all from the preferred model combinations shown in Table 1, except where specified otherwise for comparative purposes.

**Table 1:** **Model comparisons using Bayes factors calculated from marginal likelihoods in BEAST**

**Dataset**	**Preferred model combination**	**Non-preferred model combination**	**Bayes factor with interpretation**
Monocots, cpDNA			
	UCLN, BD, **p**	UCLN, BD, **np**	19510.4***
	UCLN, Yule, **p**	UCLN, Yule, **np**	19510.8***
	UCLN, **Yule**, p (best overall)	UCLN, **BD**, p	19.31***
Xanthorrhoeaceae, cpDNA			
	UCLN, BD, **np**	UCLN, BD, **p**	26.53***
	UCLN, Yule, **np**	UCLN, Yule, **p**	29.66***
	RLC, BD, **np**	RLC, BD, **p**	38.85***
	RLC, Yule, **np**	RLC, Yule, **p**	59.98***
	UCLN, **Yule**, np	UCLN, **BD**, np	8.39**
	RLC, **Yule**, np	RLC, **BD**, np	16.26***
	**RLC**, Yule, np (best overall)	**UCLN**, BD, np	7.36**
Xanthorrhoeaceae, rpb2			
	UCLN, BD, **p**	UCLN, BD, **np**	238.44***
	UCLN, Yule, **p**	UCLN, Yule, **np**	60.42***
	RLC, BD, **p**	RLC, BD, **np**	219.8***
	RLC, Yule, **p**	RLC, Yule, **np**	267.27***
	UCLN, **BD**, p	UCLN, **Yule**, np	16.08***
	RLC, **Yule**, p	RLC, **BD**, p	204.75***
	**RLC**, Yule, p (best overall)	**UCLN**, BD, p	249.98***

Within each dataset, model comparisons follow the nested sequence: partitioned (p) vs non-partitioned (np) loci; Birth-death (BD) vs Yule phylogenetic tree models; and uncorrelated lognormal (UCLN) vs random local clocks (RLC). Each row compares the bolded models, with other models held constant. For each dataset, the best model combination overall (highest marginal likelihood) is indicated, and in each case is (very) strongly preferred to the second best. Asterisks after the Bayes factors indicate their interpretation according to [27]: ** = “strong” (6 ≤ BF < 10) and *** = “very strong” (BF ≥ 10) evidence favouring the model with the higher lnL. Results are shown for path sampling only because those from stepping stone sampling were identical.

### Are substitution rates slow in *Xanthorrhoea*?

Substitution rates were compared across *Xanthorrhoea* and its outgroups using the relative rates test, local clock permutation test and results of the BEAST analyses, and all indicated a markedly slower rate in *Xanthorrhoea* than in its close relatives.

The simple relative rate test [25] does not assume a phylogenetic tree and makes pairwise comparisons of sequence dissimilarities between two groups relative to an outgroup to assess whether the two sets of dissimilarities are significantly different. We used uncorrected p-distances among sequences and found significant substitution rate differences between *Xanthorrhoea* and its sister groups (Hemerocallidoideae plus Asphodeloideae) in both loci (cpDNA and *rpb2*) and relative to both alternate outgroup sequences (*Lomandra confertiflora* and *L. glauca*): two-tailed Mann–Whitney U-test, *P* < 0.0001 in every case.

The local-clock permutation test [26] is a phylogenetic tree-based randomisation test that corrects for within-clade rate variation. We performed one-tailed LCPT tests, which showed that substitution rate has been significantly slower in *Xanthorrhoea* than in its sister groups, both in cpDNA (*P* = 0.002) and in *rpb2* (full dataset, *P* = 0.027; rarefied terminal sample, *P* = 0.029).

From the BEAST posteriors, estimated rates in cpDNA were slower in Xanthorrhoeaceae than in monocots as a whole and, within the family, slower for both loci in Xanthorrhoeoideae than in the other two subfamilies (Additional file 2: Table S2). The median substitution rate in Xanthorrhoeaceae was considerably slower in cpDNA than in *rpb2* introns, under both the UCLN (7.5 vs 69.8 × 10^−4^ substitutions/site/Myr) and RLC (6.4 vs 35.6 × 10^−4^ substitutions/site/Myr) clocks, and marginally slower than in the exons (Additional file 2: Table S2). This result was consistent with the pattern observed across plants generally (reviewed in [16,31,32]). For example, Gaut [32] estimated the synonymous rate across multiple plastid genes to be 11.3 (95% BCI = 4.5-17.7), which overlaps our estimate of 15.8 (13.5-18.3) across monocots (Additional file 2: Table S2). His estimate for nuclear genes of 60.3 (45–75) is faster than ours but this could reflect conservatism in *rpb2*, or the generally slower rates in Xanthorrhoeaceae, or that we have not distinguished synonymous from non-synonymous rates.

### Rate shifts within Xanthorrhoeaceae

The pattern of rate variation within the trees inferred by BEAST differed considerably between clock models, as expected. For both loci, under the UCLN clock most branches had a different rate from their parent (thus implying many rate shifts), though rates within *Xanthorrhoea* varied less, and were generally lower, than in the sister groups. By contrast, the RLC model inferred a small number of local clocks, with few shifts between them (Figures 6 and 7, Additional file 3: Figure S7; details below). For both loci, substitution rates inferred within the *Xanthorrhoea* crown by RLC were much lower than from UCLN, by an order of magnitude or more, and were significantly different because the 95% BCIs do not overlap (Additional file 2: Table S2 and Additional file 3: Figure S11 d-f). This result was expected following the relative rates tests. By contrast, the UCLN clock-rate differences between these taxa were neither as large nor significant, though cpDNA rates in Xanthorrhoeaceae were significantly slower than in monocots overall (Additional file 2: Table S2 and Additional file 3: Figure S11 a-c).

To test whether the number of rate shifts inferred under the RLC model using the real data departed significantly from the null expectation (zero shifts, i.e. a strict molecular clock), we ran priors-only analyses (i.e. without data) and compared overlap between the two distributions for this parameter [cf. 9]. The relaxed local clocks (RLC) model inferred 3–6 (95% BCI) substitution rate shifts in the cpDNA posterior and ≤ 3 in *rpb2* (Additional file 3: Figure S12). In both loci (and both partitions of *rpb2*), the prior expectation of a single (strict) molecular clock was rejected, as there was essentially no overlap between the 95% BCIs of the prior and posterior distributions of the “rateChangeCount” parameter, the priors being 0–1 in both loci (Additional file 3: Figure S12). Additionally, Fisher exact tests indicate that each pair of prior and posterior distributions is significantly different (*P* = 0.0000). Downward rate shifts in *Xanthorrhoea* were inferred either at the base of the stem (cpDNA, Figure 6) or within the crown (*rpb2* introns, Figure 7; *rpb2* exons, Additional file 3: Figure S7) and these low rates were sustained throughout the crown in all three partitions.

Within the *rpb2* crown of *Xanthorrhoea*, the RLC clock inferred two shifts to rates that are slower than in the stem but it is unclear whether these shifts occurred sequentially or independently (Figure 7, Additional file 3: Figure S7 and Additional file 2: Table S2). In cpDNA, one or two sequential downward rate shifts are inferred in *Xanthorrhoea*: one shift in the stem, possibly followed by a smaller shift at the crown node (Figure 6, Additional file 2: Table S2). Although neither individual shift was significant (judged by overlap between the BCIs at successive nodes), the cumulative change between the MRCA of Asphodeloideae + *Xanthorrhoea* (95% BCI = 6.1-15.6 × 10^−4^ substitutions/site/Myr) and the crown of the latter (95% BCI = 0.18-1.7 × 10^−4^ substitutions/site/Myr) was significant. Given that rate shifts can be detected only at nodes, this result is consistent with either (a) one or more incremental downward shifts along the stem of *Xanthorrhoea* (i.e. between the stem and crown node) and/or (b) independent shifts within the *Xanthorrhoea* crown.

### Age estimates within *Xanthorrhoea*

**Figure 8:**
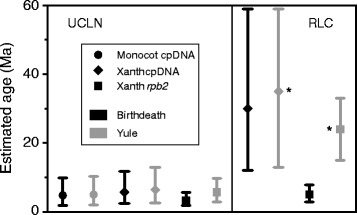
**Estimated age of the** ***Xanthorrhoea*** **crown from three datasets.** Estimates are from BEAST with UCLN and RLC clock models in combination with Birth-death and Yule speciation models. DNA substitution models were partitioned (details in text) based on the results of Bayes factor tests for each dataset. Each data point represents the median age estimate for a different combination of data and models and error bars are 95% BCIs. Asterisks mark the best combination of models for each dataset (except monocots), as indicated by Bayes factors. Abbreviation: Xanth = Xanthorrhoeaceae.

Age estimates within *Xanthorrhoea* by BEAST varied among loci and clock models, though the effect of clocks was greater (Table 2). The most significant differences occurred in the *Xanthorrhoea* crown age estimates (Figure 8). When comparing the same model across the different loci, the estimates were almost identical (Table 2; except Birth-death with RLC, see below) but the differences were large and significant between clock models. Estimates under UCLN (median ages 3–6 Ma) were much younger than those under RLC (26–30 Ma). Moreover, the 95% BCIs did not overlap between estimates from the different clocks (Figure 8). Stem-age estimates varied less between models than did crown ages (Table 2). From the Xanthorrhoeaceae-cpDNA dataset, the estimate for the *Xanthorrhoea* stem was almost identical under both clock models (58 and 59 Ma) and older in the monocot-cpDNA dataset (68 Ma), though the 95% BCIs overlap (Table 2). From *rpb2*, the median stem ages were very similar under both models and younger (40 and 44 Ma) than the estimates from cpDNA, though the BCIs overlapped (Table 2).

**Table 2:** **Ages of selected nodes estimated by BEAST**

**Dataset**	**Clock model**	**Tree model**	**Partitioned**	**Node age estimates (with 95% BCI)**				
				**Xanthorr-hoeaceae crown**	***Xanthorr-hoea*** ** stem**	***Xanthorrhoea*** ** crown**	**Hemerocall-idoideae crown**	**Asphodel-oideae crown**
Monocots, cpDNA								
	UCLN	Birth-death	no	72 (62–83)	68 (58–78)	4.8 (1.9-9.8)	62 (54–72)	35 (21–53)
	UCLN	Birth-death	yes	68 (60–78)	64 (56–72)	4.8 (2.0-9.0)	58 (51–65)	31 (20–44)
	UCLN	Yule	no	74 (63–85)	70 (60–81)	5.0 (2.0-10.3)	63 (56–75)	37 (21–55)
	**UCLN**	**Yule**	**yes**	**70 (61–79)**	**65 (58–74)**	**4.8 (2.2-9.3)**	**59 (52–67)**	**32 (20–47)**
Xanthorrhoeaceae, cpDNA								
	UCLN	Birth-death	no	71 (69–73)	58 (43–70)	5.7 (2.4-11.7)	60 (58–72)	34 (22–47)
	UCLN	Birth-death	yes	71 (70–73)	60 (49–70)	5.0 (2.4-8.7)	66 (59–72)	35 (26–45)
	UCLN	Yule	no	71 (70–73)	58 (44–70)	6.4 (2.6-13.0)	66 (59–72)	35 (23–48)
	UCLN	Yule	yes	71 (70–73)	60 (50–71)	5.4 (2.6-9.4)	66 (59–72)	36 (27–46)
	RLC	Birth-death	no	72 (70–73)	59 (46–69)	30 (12–59)	68 (59–73)	24 (16–32)
	RLC	Birth-death	yes	72 (70–74)	63 (51–72)	24 (9–58)	61 (54–72)	27 (18–34)
	**RLC**	**Yule**	**no**	**72 (70–74)**	**60 (46–70)**	**35 (13–59)**	**69 (60–73)**	**24 (17–33)**
	RLC	Yule	yes	72 (70–73)	63 (52–72)	27 (12–68)	60 (54–71)	27 (19–34)
Xanthorrhoeaceae, *rpb2*								
	UCLN	Birth-death	no	71 (69–73)	41 (24–59)	3.4 (1.5-6.1)	-	-
	UCLN	Birth-death	yes	71 (69–73)	32 (21–45)	3.3 (1.8-5.7)	-	-
	UCLN	Yule	no	71 (69–73)	20 (3–46)	13 (2–31)	-	-
	UCLN	Yule	yes	71 (70–73)	26 (14–39)	5.7 (2.9-9.7)	-	-
	RLC	Birth-death	no	71 (69–73)	40 (31–54)	27 (17–37)	-	-
	RLC	Birth-death	yes	71 (69–73)	35 (28–43)	5.0 (2.8-7.8)	-	-
	RLC	Yule	no	71 (69–73)	40 (32–48)	26 (17–35)	-	-
	**RLC**	**Yule**	**yes**	**71 (70–73)**	**38 (30–46)**	**24 (15–32)**	**-**	**-**

The *Xanthorrhoea* crown was the target node for dating, being the broom clade with no internal calibration. In the two Xanthorrhoeaceae datasets (cpDNA and *rpb2*), the root (= family crown node) was calibrated with a normal prior, mean = 71, SD = 1.0. Rows with bolded text have the best-fit model combination for each dataset as assessed by Bayes factors using marginal likelihoods estimated from BEAST using path and stepping-stone sampling. BCI = 95% Bayesian confidence internal. UCLN = uncorrelated lognormal clock. RLC = random local clocks.

Against the general trend, the age estimated for the *Xanthorrhoea* crown under RLC with Birth-death for *rpb2* (5.0 Ma, 95% BCI = 2.8-7.8) was much younger than the other RLC estimates and within the range of estimates from UCLN (Figure 8, Table 2, Additional file 3: Figures S8 and S9). This reconstruction infers a downward rate shift in the *Xanthorrhoea* stem that remains unchanged throughout the crown for the introns (Additional file 3: Figure S8), and a single rate throughout Xanthorrhoeaceae for the exons (Additional file 3: Figure S9). However, it should be noted that the Yule model was very strongly preferred to Birth-death for *rpb2* (Table 1) and gave an age estimate well within the range of other RLC estimates for this node (24 Ma, 95% BCI = 15–32; (Table 2).

## Discussion

### Node age estimates differ significantly between uncorrelated and correlated clock models

Our BEAST analyses of the simulated datasets returned accurate dates under the RLC clock whereas UCLN invariably failed to retrieve the true date, even within the 95% BCIs. Using a single simulated tree, Dornburg et al. [9] also obtained more accurate age estimates with RLC than UCLN. With the latter clock, their estimates were misleadingly precise, i.e. ULCN gave narrower BCIs than did RLC, but was less accurate. The simulations presented here go further than [9] by using multiple simulated datasets with different tree sizes and relative clade sizes, and by explicitly testing the accuracy of age estimation in uncalibrated “broom” clades. Thus we have shown that accurate age estimates depend upon accurate clock modelling, and that these results are not an artefact of a particular tree shape or relative clade sizes. We conclude that, for data predicted to include local clocks, dating with RLC is much more likely to be accurate than with UCLN.

Median age estimates within Xanthorrhoeaceae differ by an order of magnitude under the two clock models (Table 2). This is the direct consequence of the differences in substitution rates inferred under each model. The most striking differences lie in estimates for the *Xanthorrhoea* crown: the median ages vary from 3.3 to 35 Ma and, with a single exception (discussed below), the 95% BCIs do not overlap between the different clocks for a given locus and phylogenetic tree model combination (Figure 8). In contrast, crown age estimates from different loci under the same clock are close (Figure 8), and the BCIs overlap substantially. Median age estimates also differ between clocks, by 10–20 Ma, for the *Xanthorrhoea* stem and the Asphodeloideae crown (Table 2). As the Bayes factor tests indicated a clear preference (BF = “very strong”) for the RLC model over UCLN, combined with the Yule tree model, it appears likely that the age of the *Xanthorrhoea* crown is about 24 (*rpb2*) to 35 (cpDNA) Ma with a wide range of uncertainty (inclusive 95% BCIs = 13–59).

### Where RLC locates rate shifts influences dates

In both the simulated and empirical datasets, a relatively small number of rate shifts, with rate conservation along lineages between shifts, are inferred by RLC. However, the exact number of rate shifts under RLC (Additional file 3: Figure S12), and their location on the phylogeny, are usually uncertain. Even when it appears clear that a rate shift has occurred between a parent node and its immediate daughter, the shift could have occurred anywhere along the branch connecting these nodes. This uncertainty is greater in longer branches, e.g., the stem of *Xanthorrhoea*. Another source of uncertainty is whether a net directional rate shift in a clade has occurred in a single large step or cumulatively in several smaller steps along successive branches. Such uncertainty can critically affect dating of a particular node. For example, an anomalously young date (~5.0 Ma) was estimated for the *Xanthorrhoea* crown node using RLC with a Birth-death speciation prior from the *rpb2* dataset (Table 2, Figure 8). This analysis reconstructed a single downward rate shift at the stem node (Additional file 3: Figure S8) but, in contrast, RLC with Yule reconstructed two smaller shifts, both within the crown (Figure 7) and, consequently, a much older age (~24 Ma) for the crown node (Table 2, Figure 8). Thus, the RLC clock, even if appropriate for the data, does not necessarily reconstruct dates accurately (the two reconstructions are mutually exclusive).

As the combination of Yule with RLC was very strongly preferred to Birth-death + RLC by the marginal likelihood tests, the anomalously young date from the latter combination could be discounted in this case. Nevertheless, the question remains of why the Birth-death and Yule models returned a large difference in age estimates for the *Xanthorrhoea* crown and reconstructed rate shifts that were similar but at different nodes. Perhaps the difference resulted from an interaction between the phylogenetic-branching and rate-shift parameters along the stem of *Xanthorrhoea* (a long branch). It could have arisen from mis-specification of the phylogenetic branching model, a known issue [33,34], because the only difference between these two analyses was the use of the alternative phylogenetic branching models.

### Substitution rates have slowed in *Xanthorrhoea*

We have found strong evidence in favour of a lineage effect [18] in substitution rates within Xanthorrhoeaceae. That is, the molecular clock ticks much more slowly in *Xanthorrhoea* than in the rest of the family in general. Multiple lines of evidence support these conclusions: (a) significant results from both relative rates tests (RRT and LCPT); (b) rejection of a single clock across the family by comparing the prior versus posterior estimates of the number of rate shifts under the RLC (local clocks) model; (c) inference by BEAST of downward rate shifts, of about an order of magnitude, in the stem and/or crown of *Xanthorrhoea*; and (d) replication of these findings in two loci from genomes (chloroplast and nuclear) with different modes of inheritance.

### Life history and substitution rates

Given that two unlinked loci in different genomes show the downward rate shift in *Xanthorrhoea*, the likely cause is a factor that affects both genomes rather than locus-specific factors such as selection, or genome-specific effects such as polymerase error rate or efficiency of DNA repair [16]. Instead, the concerted rate shifts in these unlinked loci suggest a factor external to the genomes, such as life-history, speciation rate, or environmental factors that affect the metabolic rate [17,18]. Given that *Xanthorrhoea* occurs across a wide range of latitude and moisture regimes, and co-occurs with multiple species from its sister groups, environment is unlikely to be the cause of the rate shift. Our results are consistent with previous studies that found that plant lineages with life history traits similar to *Xanthorrhoea* also have slow rates of molecular evolution. These traits include an arborescent palm-like life form, longevity, slow growth rate and single or few-branched stems. The palm-like life form has one or few apical meristems and is likely to have a slower overall rate of mitosis than much-branched plants with many growing tips. Our results are also consistent with earlier findings that speciation rate and substitution rate are positively correlated, though the causal factors are unclear [18,35,36]: *Xanthorrhoea* has fewer species (28) and a slower rate of substitution than its sister groups (Hemerocallidoideae ~ 85, Asphodeloideae ~ 785) [37].

Within *Xanthorrhoea*, rates appear to vary with growth habit: *X. macronema* and *X. gracilis* have *rpb2* substitution rates at least twice as fast as in other grasstrees (Additional file 2: Table S2) and differ from them in being trunkless, softer and grass-like. *Xanthorrhoea gracilis* is shallow-rooted and can be killed by fire more readily than the other species [21]. However, the underlying factors linking multiple correlated life-history traits to molecular evolutionary rates remain unclear. As fewer-branched plants have fewer apical meristems, the mitosis hypothesis [20], which posits that substitution rate is correlated with the number of mitotic events, could be tested by comparing substitution rates among plant lineages that differ in their degree of branching but have otherwise similar life histories.

### Implications for macroevolution

The possibility that UCLN significantly under-estimates the age of broom clades has implications for macroevolutionary investigations using molecular dating. For example, in *Xanthorrhoea*, the RLC model preferred by our analyses suggests a crown age of 24–35 Ma, implying that *Xanthorrhoea* diversified from about the end of the Eocene. The broom crown of this clade, combined with the long stem, suggests that an extinction event [cf. 1] occurred at this time, when global climate deteriorated significantly, and then the clade re-diversified through the Oligocene and Miocene. This timing and diversification pattern are congruent with those in other taxa co-occurring with *Xanthorrhoea* in the Australian temperate sclerophyll biome, such as *Callitris* (Cupressaceae), Casuarinaceae, Eucalypteae (Myrtaceae), and pea-flowered legumes (Fabaceae: Bossiaeeae), which are considered to have responded to the same climate-change event [38]. In contrast, the much younger crown ages obtained using UCLN for *Xanthorrhoea* (3–6 Ma), but rejected by our tests, would favour a conclusion that extinction and re-radiation occurred during the Pliocene and Pleistocene. This timing does not coincide with the similar diversification shifts in the other taxa.

Other areas of investigation might also be biased by clock model mis-specification. For example, vicariance hypotheses for the origins of the New Caledonian biota have been rejected after divergence times for multiple clades with sisters in other landmasses were found to be too young compared with geological separation [39]. Similarly, claims that New Zealand drowned completely during the Oligocene have gained support from clade-age estimates that post-date the geological emergence of the islands as subaerial land [40]. The age of any such clades that were broom-shaped and dated using UCLN should be reassessed using RLC and marginal likelihood model comparisons.

## Conclusion

We have shown that large dating errors can result from the UCLN clock model (compared with RLC) if its assumptions are violated. Neither clock is necessarily superior in general: model choice should be contingent upon how well a given dataset meets its assumptions. In datasets with clade-specific substitution rates, the RLC model provides a significantly more accurate approach than the UCLN model, especially in long-branch clades that lack calibrations. We found significant differences in age estimates using these models with such datasets, whether simulated or empirical (*Xanthorrhoea*). In simulated rate-heterogeneous phylogenies with a slowly evolving “broom” clade, the uncorrelated clocks model always significantly underestimated the true age of the broom crown, whereas the random local clocks model consistently succeeded. UCLN likely underestimates the age of uncalibrated broom clades because the uncorrelated model is poor at detecting rate shifts [9]. Calibration of the broom can reduce this dating error but it does not correct model mis-specification [9]. Therefore, it is important to compare the suitability of both models by using *a priori* criteria (e.g., life history, relative-rates test, branch length variation in a phylogram) or *a posteriori* marginal likelihood tests. More extensive studies of clock models are needed to determine their applicability to different kinds of datasets [10]. For instance, local clocks and autocorrelated clocks are similar in principle but differ in the number of rate shifts allowed and we did not compare these clocks in this study.

In analyses of *Xanthorrhoea*, the two clock models led to very different conclusions about the evolution of the genus: rapid diversification with severe aridification in the Pliocene/Pleistocene (UCLN) or diversification following the onset of global cooling and drying in the Miocene (RLC). Life history traits of *Xanthorrhoea* suggested that the substitution rate might be slower than in its sister groups, and use of the RLC clock was supported by relative rate testing and marginal likelihood tests. The older crown age (24–35 Ma) estimated for *Xanthorrhoea* by the preferred RLC model is consistent with re-diversification following the end-Eocene extinctions, as inferred in co-occurring taxa and attributed to global climate change.

A challenge for the future will be to determine the timing of substitution rate shifts more accurately because uncertainty can severely affect dating. Currently, resolution of this uncertainty requires independent evidence, such as fossil-based calibration at critical nodes, e.g. in broom clades.

## Methods

In order to develop a biologically realistic simulation, we first analyzed the *Xanthorrhoeaceae* dataset so that we could use it as a basis for choosing parameter values. The methods used for the empirical data analyses are described after those of the simulations.

### Simulations of strong among-clade substitution rate variation

To test the accuracy of the RLC and UCLN models in retrieving the known age of an uncalibrated crown node with a long stem, we simulated data with parameters that reflected the critically relevant properties of the *Xanthorrhoeaceae* dataset.

All simulations were performed in the R software environment [41]. Using the R package TESS [42], we simulated 100 ultrametric trees under a Birth-death model of phylogenetic branching (i.e., a stochastic, equal rates Markov model with speciation and extinction rate parameters). The distribution of branch lengths across the simulated trees was a function of the specified speciation and extinction rates. Each tree was composed of two primary clades: a “broom” clade [1] with a long stem (45–25 Ma) and recent crown (25 Ma), and a “bush” clade with a short stem (45–40 Ma) and old crown (40 Ma). From this set of 100 trees, we chose 10 with fewer than 50 terminal branches in total, and with the proportion of branches in each clade varying among trees (Additional file 2: Table S1).

Next, using the R package PhyloSim [43], we simulated the evolution of a 1 Kb DNA data set over each of these trees. We used a GTR + Γ model of substitution, with the alpha parameter set to 1. In the commonly used Markov model of DNA evolution, substitution rates are modelled with parameters denoting the probability of particular kinds of substitution over an infinitesimal time interval – the higher the probability of a substitution, the more frequently it occurs. In our simulations, we used a base GTR model with the following parameters: μTC = 1, μTA = 2, μTG = 3, μCA = 1, μCG = 2, and μAG = 3. Base frequencies were set to T = C = 0.667 and A = G = 0.333. The scaling parameter on the Q matrix was an order of magnitude slower in the broom clade (0.001) than in the bush clade (0.01); this ratio was close to what we estimated from the empirical data. Thus, for μAG, the substitution probability in the broom clade was 3 × 0.001 = 0.003, conditioned on base frequencies and the Γ distribution of among-site rate heterogeneity.

Phylogenetic divergence times were estimated using BEAST v1.8.0 for each simulated DNA dataset under RLC and UCLN models of among-lineage substitution rate variation, and under Birth-death and Yule phylogenetic tree models. Each estimate assumed a general time-reversible plus Gamma (GTR + Γ) substitution model and used three log-normal stem-node age priors: one was placed at the stem-node of the broom clade and two were placed at the stem-nodes of each of two clades internal to the bush clade (to match the real data). The mean and offset of each calibration prior were equal to half the distance between the crown and stem nodes of the respective clade (Figure 1). MCMC analyses were run in BEAST for 50 to 200 M generations, sampling parameters and trees every 20 K generations. Parameter traces were viewed in Tracer v1.6 [44] to determine when each analysis began to sample from the stationary distribution. Trees sampled before stationarity had been reached (burnin) were discarded. Bayes factors [27] calculated from the marginal likelihoods using path sampling and stepping-stone sampling [45,46] were used to evaluate the fit of RLC and UCLN models to each simulated dataset. All R scripts, simulated trees, and BEAST xml files and annotated maximum clade credibility trees are provided as Appendix 1 in [47].

### Xanthorrhoeaceae: sampling, DNA sequencing and alignment

Of the 28 named species of *Xanthorrhoea* [48], we sampled 25 with at least one accession, plus outgroups representing the other two subfamilies of Xanthorrhoeaceae and some outgroups from Asparagales (Appendices 2–3 in [47]). Vouchers are lodged at the Australian National Herbarium (CANB).

Genomic DNA was extracted from silica-gel-dried leaf tissue using a DNeasy plant mini kit (Qiagen) according to the manufacturer’s protocol. PCR was conducted using 25μl reaction volumes containing 2.0 mM MgCl_2_, 0.2 mM of each dNTP, 0.2 μM primer, 0.75 units PlatinumTaq (Invitrogen) and 2μl of the template DNA. The DNA regions amplified were from the chloroplast (*ndhF* and *trnL-trnF*; “Xanthorrhoeaceae-cpDNA” dataset) and nuclear *rpb2* (“Xanthorrhoeaceae-*rpb2*” dataset). We sometimes refer to these two datasets collectively as the “Xanthorrhoeaceae-only” datasets. The primers used for amplification were 1252f and Mel-r1 [49] for *ndhF*, trnL-F c and trnL-F f [50] for *trnL-trnF*, and P7F [51] with ex22r [49] for *rpb2*. The resulting products were sequenced in both directions using ABI Big Dye 3.1 chemistry on an ABI Prism 3100 genetic analyser (Applied Biosystems, Foster City, California). Sequences obtained were edited using Sequencher 4.5 (GeneCodes), aligned initially using MAFFT [52] in CIPRES [53] and manually adjusted in Se-Al v2.0a11 [54].

Direct sequencing of many of the nuclear *rpb2* fragments revealed the presence of polymorphic sites and indels that confounded unequivocal reading of the sequence. To isolate individual copies from these polymorphic sequences the PCR products were run out on 2% TAE agarose gel, excised and then purified using BRESAclean/Mobio Quickclean 300 kit (GeneWorks). The purified products were then cloned using the pGEM-T Vector System II (Promega) with the manufacturer’s protocol at 1/8th volume. Individual colonies were amplified by PCR using the same protocol as with the original *rpb2* products. Five colonies per original product were sequenced and these were used to compare with the original “parental” sequence to attempt to distinguish between substitutions resulting from Taq error and those that were suspected polymorphic bases in the parent sequence (see [49] for a discussion of this procedure).

Within *Xanthorrhoea*, there were only 12 cpDNA haplotypes, i.e., multiple individuals and species shared identical haplotypes, and the dataset was pruned to a single terminal per haplotype for the analyses. Cloning of *rpb2* yielded many more unique haplotypes than species but did not appear to comprise multiple loci predating the origin of *Xanthorrhoea* because the genus was monophyletic. If locus duplications had predated divergence of *Xanthorrhoea* from its sister group(s), then we would expect to see two or more subtrees (representing the duplicated loci), each including *Xanthorrhoea* and any sister group that also inherited both copies of the duplicated locus, i.e. *Xanthorrhoea* would appear paraphyletic [55]. We rarefied the *rpb2* haplotype sample to minimise the node density artefact in substitution rate modelling [56] because not all species were sampled in the other subfamilies. In the rarefied sample, we represented the diversity both of species and of putative paralogs by sampling from all deeper-level clades. Analyses using both the full and rarefied datasets were compared and, as the results did not differ, usually only those from the rarefied data are reported here. Even after rarefaction, species-level sampling was greater in *Xanthorrhoea* than in the sister groups. This makes our hypothesis test conservative because the node density artefact tends to inflate the estimate of molecular evolutionary rate in lineages with more nodes [56]; thus, our estimates of rates in *Xanthorrhoea* are likely to err on the high side, relative to the less well-sampled sister groups.

As there is no fossil-based calibration available for the Xanthorrhoeaceae crown node, we made a robust age estimate to use as a prior in our dating analyses within the family. We created a third dataset (“Monocots-cpDNA”) of sequences downloaded from Genbank of the cpDNA regions that we have sequenced for species of *Xanthorrhoea* (*ndhF* and *trnL-trnF*) for 125 taxa representing all major clades within monocots (Appendices 2–3 in [47]), including *Acorus* for the root [37], and aligned these with the sequences obtained in this study.

### Xanthorrhoeaceae: phylogenetics and relaxed molecular clock dating

Phylogenetic relationships were inferred using maximum likelihood with a general time-reversible (GTR) + Γ model implemented in RAxML ver. 7.4.2 [57] on CIPRES [53]. DNA sequences were partitioned with separate substitution models set for the exons of *ndhF* and *rpb2* (SRD06 model) and for the noncoding regions (introns and spacers combined) of each locus (cpDNA and *rpb2*/nDNA).

Time-calibrated phylogenies were inferred using BEAST v1.8.0 [58] with uncorrelated lognormal (UCLN) and random local clocks (RLC) clock models. Each partition (defined as above) was given a separate clock model. For comparison, two phylogenetic branching models were used: a calibrated Yule model [59] and a Birth-death model with incomplete sampling [60]. Both partitioned and non-partitioned models were run as described above for the RAxML analyses. Relative fit of the alternative models for sequence partitioning, tree priors and clock models was evaluated using marginal likelihoods obtained by path sampling and stepping-stone sampling [45,46] to calculate Bayes factors [27]. To ensure stationarity and convergence of the Bayesian MCMC chains, two or more parallel runs were made and Tracer v1.6 [44] used to check that the post-burn-in effective sample sizes in the combined logs were > 200 for all parameters. Run length was a minimum of 100 million generations and was increased up to 300 million if needed to achieve stationarity and convergence. To display the results, maximum clade credibility trees were annotated in FigTree v1.4.0 [61], as recommended by the author.

### Xanthorrhoeaceae: calibration

The crown node of monocots (MRCA of *Acorus* and the rest), and its daughter node (Alismatales vs the rest) were constrained with normal priors (respectively, mean = 145 Ma, SD = 1.0 and mean = 134 Ma, SD = 1.0), that encompass two very similar age estimates [62,63] for these nodes (nodes 1–2 in Additional file 3: Figure S2). These secondary calibrations were given normally distributed priors, whereas the following fossil-based calibrations were given lognormal priors, as recommended in each case [6]. The MRCA of *Spathiphyllum*, *Monstera* and *Arisaema* (Araceae, node 3) was constrained with an offset (minimum age) = 115 Ma and SD = 1.0), based on the co-occurrence of diagnostic fossil pollens of *Spathiphyllum* and Monstereae in Portugal [64]. Although the optimal location of these fossils on the phylogeny remains uncertain [65,66], our placement is within the likely range accepted by these authors. A constraint at the MRCA of palms (node 4: offset = 85.8, SD = 1.0) was derived from the earliest known palm fossil *Sabalites carolinensis* [67]. The stem node of Poales (node 5: offset 115 = Ma, SD = 1.0) was constrained based on the earliest known fossils of Poales [68] and a similar secondary estimate [69]. Calibration of node 6 (offset = 45.0, SD = 1.0) was placed at the MRCA of *Dianella* and *Phormium* (“phormioid” clade), which share leaves that are isobifacial and equitant immediately above the sheath but distally become dorsiventrally flattened, whereas *Pasithea*, sister to this clade, lacks an isobifacial zone [37]. The clade was calibrated with an Eocene-age *Dianella*-like fossil [70]. In the analyses of the smaller, Xanthorrhoeaceae-only datasets, the root (= crown node of the family) was given a normal prior of 71.2 (SD = 1.0), being the midpoint of the range of mean estimates (68–74) from the BEAST analyses of the larger (monocots) dataset (Table 2).

### Have substitution rates slowed in *Xanthorrhoea*?

Substitution rates were compared across *Xanthorrhoea* and its outgroups, using the relative rates test, local clock permutation test, and rate estimates from the BEAST posteriors.

We first used the standard relative-rates test [71] to assess whether the substitution rate in *Xanthorrhoea* is significantly different from that in the rest of the family. This test has the advantage of simplicity: it does not assume a phylogeny and makes pairwise comparisons of sequence dissimilarities between two groups (*Xanthorrhoea* and the other subfamilies) and an outgroup (*Lomandra confertiflora* and *L. glauca* from Asparagaceae) to assess whether the two sets of dissimilarities are significantly different. We used both the other subfamilies simultaneously for the rate comparisons with *Xanthorrhoea* for the following reasons. Xanthorrhoeaceae comprises three clades, which are *Xanthorrhoea* (= subfam. Xanthorrhoeoideae) and its two ister groups, subfamilies Hemerocallidoideae and Asphodeloideae. Monophyly of the family and of each subfamily is well supported by our analyses of cpDNA across all monocots (Additional file 3: Figures S2 and S3) and by other recent studies [28-30]. However, the relationship among the subfamilies has been uncertain (reviewed in [37]), though all the above analyses have favoured (Asphodeloideae, (Hemerocallidoideae, Xanthorrhoeoideae)). This result was also obtained from the Xanthorrhoeaceae-*rpb2* dataset (Additional file 3: Figure S4) but in the Xanthorrhoeaceae-cpDNA dataset *Xanthorrhoea* was sister to Asphodeloideae (Additional file 3: Figure S5).

We implemented the relative rates test using uncorrected proportional distance (“p-distance”) as the dissimilarity measure to compare all ingroup-outgroup sequence pairs. The standard test uses a likelihood ratio test based on the variances of the two sets of pairwise distances [71] but is problematic because it can return a significant difference that reflects rate variation within lineages (groups) alone [26]. We avoided this problem using a two-tailed Mann–Whitney U test [72], which makes no assumption about variance, and simply tests the null hypothesis that one set of distances (from group 1 to the outgroup) is stochastically neither larger nor smaller than the other set (from group 2 to the outgroup). Four tests were done using each locus (cpDNA and *rpb2*) in turn, and with each outgroup (*Lomandra* species) in turn.

We alternatively used the local-clock permutation test [26], which is phylogeny-based, unlike the relative rates test. The local-clock permutation test calculates the log likelihood difference (Δ) between a single (global) clock model and a local (within-lineage) clocks model for comparison with a null distribution generated by randomising the tips across the lineages [26]. If Δ calculated from the original (unpermuted) data is significantly greater than that calculated from the permuted data (i.e., falls outside the 5% tail of the null distribution), then the null hypothesis of a single global clock is rejected. We implemented this test using the protocol and scripts developed by [26] in combination with PAML 4.4b 71], with 1000 permutations, as recommended. We tested the *rpb2* and cpDNA datasets separately.

### Availability of supporting data

The data sets supporting the results of this article are available in the Dryad repository, http://dx.doi.org/10.5061/dryad.d1nb6.

## Additional files

Additional file 1: Figure S1. Mass post-fire flowering in a population of *Xanthorrhoea thorntonii* in Central Australia. Additional file 2: Table S1. Simulations of strong among-clade substitution rate variation: estimates of age of broom crown node. **Table S2.** Clock rates in substitutions/site/Myr x 10^−4^ estimated by BEAST under each clock model for selected clades. Additional file 3: Figure S2. Maximum likelihood phylogeny (best RAxML tree) of monocots including Xanthorrhoeaceae. **Figure S3.** Uncorrelated lognormal clock (UCLN) chronogram of monocots including Xanthorrhoeaceae (bottom of page, with subfamilies labelled) and derived from combined partitioned *ndhF* and *trnL-trnF* (chloroplast DNA) sequences using BEAST with a Yule tree model. **Figure S4.** Maximum likelihood phylogeny of Xanthorrhoeaceae, derived using RAxML from combined partitioned *ndhF* and *trnL-trnF* (chloroplast DNA) sequences, showing branch lengths proportional to substitutions per site. **Figure S5.** Maximum likelihood phylogeny of Xanthorrhoeaceae, derived using RAxML from combined *ndhF* and *trnL-trnF* (cpDNA) sequences, showing branch lengths proportional to substitutions per site. **Figure S6.** UCLN chronogram of Xanthorrhoeaceae derived from combined *ndhF* and *trnL-trnF* (cpDNA) using BEAST with a Yule tree model. **Figure S7.** RLC chronogram of Xanthorrhoeaceae derived from sequences of *rpb2* (nuclear DNA) using BEAST with Yule tree model. **Figure S8.** RLC chronogram of Xanthorrhoeaceae derived from sequences of *rpb2* (nuclear DNA) using BEAST with a Birth-death tree model. **Figure S9.** RLC chronogram of Xanthorrhoeaceae derived from sequences of *rpb2* (nuclear DNA) using BEAST with a Birth-death tree model. **Figure S10.** UCLN chronogram of Xanthorrhoeaceae derived from sequences of *rpb2* (nDNA) using BEAST with a Yule tree model. **Figure S11.** Estimates of DNA substitution rates in monocots, *Xanthorrhoea* and its two sister groups using BEAST for all three datasets under both alternative clock models and the Yule tree-growth model. **Figure S12.** Prior and posterior distributions of number of rate shifts inferred by RLC clock analyses using BEAST from cpDNA and *rpb2* sequences.

## Abbreviations

UCLN: Uncorrelated lognormal molecular clock model
RLC: Random local clocks model
BCI: Bayesian confidence interval
GTR + Γ: General Time Reversible plus Gamma (DNA substitution model)
