# Supplementary figures and images for: Clock model makes a large difference to age estimates of long-stemmed clades with no internal calibration: a test using Australian grasstrees
Source: BMC Evol Biol. 2014 Dec 19;14:263. doi: 10.1186/s12862-014-0263-3 (PMC4279595; doi:10.1186/s12862-014-0263-3)

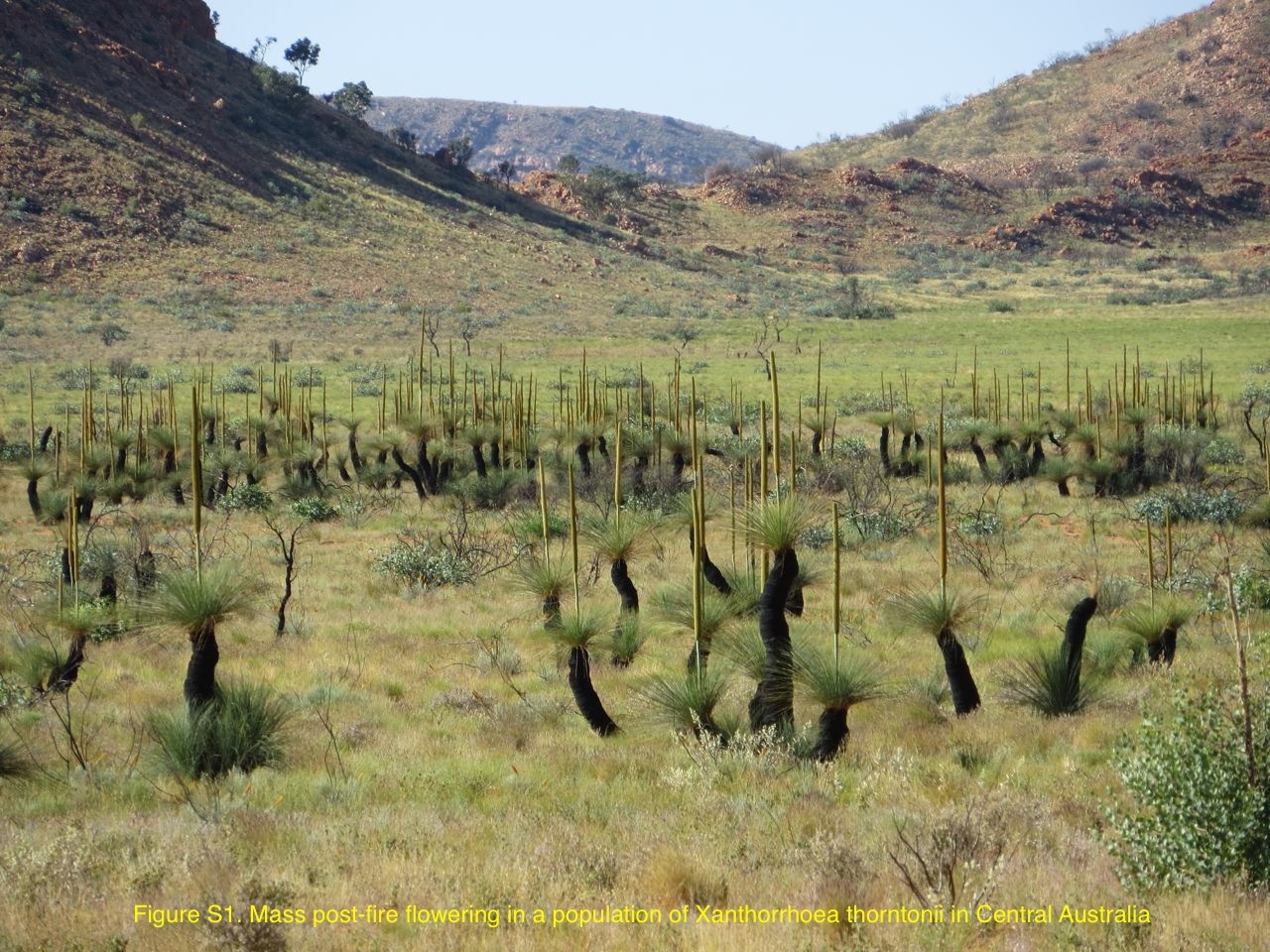

Supplement: Additional file 1: Figure S1. — Mass post-fire flowering in a population of Xanthorrhoea thorntonii in Central Australia. [file 12862_2014_263_MOESM1_ESM.jpeg]
