# Supplementary material for: Clock model makes a large difference to age estimates of long-stemmed clades with no internal calibration: a test using Australian grasstrees
Source: BMC Evol Biol. 2014 Dec 19;14:263. doi: 10.1186/s12862-014-0263-3 (PMC4279595; doi:10.1186/s12862-014-0263-3)
Supplement: Additional file 2: Table S1. — Simulations of strong among-clade substitution rate variation: estimates of age of broom crown node. Table S2. Clock rates in substitutions/site/Myr x 10−4 estimated by BEAST under each clock model for selected clades. [file 12862_2014_263_MOESM2_ESM.pdf]

Table S1. Simulations of strong among-clade substitution rate variation: estimates of age of broom crown node. The 'true' (simulated) age of the broom crown node is 25 Ma. The upper and lower values represent the 95% BCIs from BEAST.

	Uncorrelated lognormal (UCLN) clock			Random local clocks (RLC)			Tree size (#tips)		
Birth-death model	Mean	Lower	Upper	Mean	Lower	Upper	Bush	Broom	Tree
Tree 1	4.3898	3.3359	5.8821	21.3570	12.5507	34.8548	21	8	29
2	4.9736	2.9500	7.6157	30.1473	19.0482	41.8640	13	17	30
3	3.8854	2.1107	5.8598	30.1382	19.8739	38.5784	10	22	32
4	4.0507	2.6852	5.6188	31.2605	19.4952	40.2527	10	23	33
5	3.596	0.9990	7.4140	29.3506	17.9086	38.2796	31	4	35
6	4.6116	1.8907	11.3034	29.4219	16.4058	42.2400	11	11	22
7	4.8263	2.0299	11.0917	29.3311	16.3736	40.0179	6	13	19
8	3.1049	1.8206	4.4384	20.9054	11.3086	34.0304	6	20	26
9	3.7666	2.7397	4.9016	32.2281	21.2457	39.5230	14	28	42
10	3.9043	2.0976	5.8400	29.6382	19.0891	37.8991	10	21	31
Mean all trees	4.1109	2.2659	6.9966	28.3778	17.3299	38.7540			
Yule model									
Tree 1	4.4294	3.2375	5.7842	27.9442	14.8074	41.0589	21	8	29
2	5.4678	3.5517	8.0696	34.9647	23.3738	43.3503	13	17	30
3	4.8769	2.8928	8.0410	33.5540	23.7047	39.6243	10	22	32
4	4.5256	3.2146	6.2645	28.8422	18.6666	38.1780	10	23	33
5	3.6056	1.2237	7.9621	28.9050	17.8108	35.5290	31	4	35
6	5.9209	2.3499	17.5212	31.0299	18.7476	43.0976	11	11	22
7	6.6250	2.4529	24.8610	25.2272	14.8151	37.2683	6	13	19
8	4.0330	2.6144	6.1761	25.0006	14.9336	36.9937	6	20	26
9	4.3842	3.3293	5.7383	28.3256	19.0340	37.1197	14	28	42
10	5.2411	2.6502	13.6818	30.3407	20.6046	38.0061	10	21	31
Mean all trees	4.9110	2.7517	10.4100	29.4134	18.6498	39.0226			

Table S2. Clock rates in substitutions/site/Myr x 10^{-4}: Mean (95% BCI) estimated by BEAST under each clock model for selected clades. Rates for whole trees (monocots and Xanthorrhoeaceae) are values of the 'meanRate' parameter and its 95% HPD calculated in Tracer from the BEAST log file (post-burnin) under the preferred models for tree-growth (Yule in every case), and estimated separately for each clock and each partition (where BF tests supported partitioning). Rates for selected clades within Xanthorrhoeaceae (remaining rows of table) are the mean and 95% confidence interval of the 'rate median' parameter values across all branches within the target clade, as plotted on the annotated maximum clade credibility tree using FigTree v1.4.0 (Rambaut 2012). As *Xanthorrhoea* stem is a single node, its rate median value and 95% HPD values are presented instead. There are no data for the RLC clock from the Monocots-cpDNA dataset because the analyses did not reach stationarity.

Dataset	Monocots-cpDNA		Xanthorrhoeaceae-cpDNA		Xanthorrhoeaceae-*rpb2*			
Clock	UCLN	UCLN	UCLN	RLC	UCLN		RLC	
Partition	*ndhF*	noncoding	all	all	exons	introns	exons	introns
Monocots	10.7 (9.7-11.7)	15.8 (13.5-18.3)	-	-	-	-	-	-
Xanthorr-hoeaceae	-	-	7.5 (6.3-8.7)	6.4 (5.1-7.7)	15.4 (10.7-20.9)	69.8 (50.7-88.6)	8.2 (5.8-10.9)	35.6 (25.8-45.2)
Xanthorrhoea stem	-	-	2.1 (1.1-3.5)	2.0 (0.2-14.3)	15.0 (7.4-27.1)	53.4 (21.2-107.2)	15.2 (11.5-19.5)	70.7 (58.3-85.7)
Xanthorrhoea crown	-	-	5.0 (4.7-5.4)	0.69 (0.68-0.70)	14.2 (14.1-14.2)	24.0 (22.6-25.4)	1.2 (1.0-1.3)	3.2 (2.8-3.6)
X. gracilis + *X. macronema*	-	-	-	-	14.2 (13.8-14.5)	27.2 (21.4-33.0)	3.5 (2.9-4.1)	8.9 (8.9-8.9)
Rest of *Xanthorrhoea*	-	-	-	-	14.2 (14.1-14.3)	23.7 (22.2-25.2)	0.98 (0.98-0.99)	2.7 (2.7-2.7)
Hemerocallid-oideae	-	-	6.8 (5.9-7.8)	6.7 (6.5-7.0)	14.9 (14.1-15.7)	53.2 (20.8-85.7)	15.2 (15.2-15.2)	70.7 (70.7-70.7)
Asphodeloideae	-	-	9.0 (6.2-11.8)	12.9 (11.8-14.0)	14.9 (14.2-15.7)	72.8 (23.5-122.2)	15.5 (15.2-15.8)	71.3 (71.1-71.5)
