# Supplementary material for: Clock model makes a large difference to age estimates of long-stemmed clades with no internal calibration: a test using Australian grasstrees
Source: BMC Evol Biol. 2014 Dec 19;14:263. doi: 10.1186/s12862-014-0263-3 (PMC4279595; doi:10.1186/s12862-014-0263-3)
Supplement: Additional file 3: Figure S2. — Maximum likelihood phylogeny (best RAxML tree) of monocots including Xanthorrhoeaceae. Figure S3. Uncorrelated lognormal clock (UCLN) chronogram of monocots including Xanthorrhoeaceae (bottom of page, with subfamilies labelled) and derived from combined partitioned ndhF and trnL-trnF (chloroplast DNA) sequences using BEAST with a Yule tree model. Figure S4. Maximum likelihood phylogeny of Xanthorrhoeaceae, derived using RAxML from combined partitioned ndhF and trnL-trnF (chloroplast DNA) sequences, showing branch lengths proportional to substitutions per site. Figure S5. Maximum likelihood phylogeny of Xanthorrhoeaceae, derived using RAxML from combined ndhF and trnL-trnF (cpDNA) sequences, showing branch lengths proportional to substitutions per site. Figure S6. UCLN chronogram of Xanthorrhoeaceae derived from combined ndhF and trnL-trnF (cpDNA) using BEAST with a Yule tree model. Figure S7. RLC chronogram of Xanthorrhoeaceae derived from sequences of rpb2 (nuclear DNA) using BEAST with Yule tree model. Figure S8. RLC chronogram of Xanthorrhoeaceae derived from sequences of rpb2 (nuclear DNA) using BEAST with a Birth-death tree model. Figure S9. RLC chronogram of Xanthorrhoeaceae derived from sequences of rpb2 (nuclear DNA) using BEAST with a Birth-death tree model. Figure S10. UCLN chronogram of Xanthorrhoeaceae derived from sequences of rpb2 (nDNA) using BEAST with a Yule tree model. Figure S11. Estimates of DNA substitution rates in monocots, Xanthorrhoea and its two sister groups using BEAST for all three datasets under both alternative clock models and the Yule tree-growth model. Figure S12. Prior and posterior distributions of number of rate shifts inferred by RLC clock analyses using BEAST from cpDNA and rpb2 sequences. [file 12862_2014_263_MOESM3_ESM.pdf]

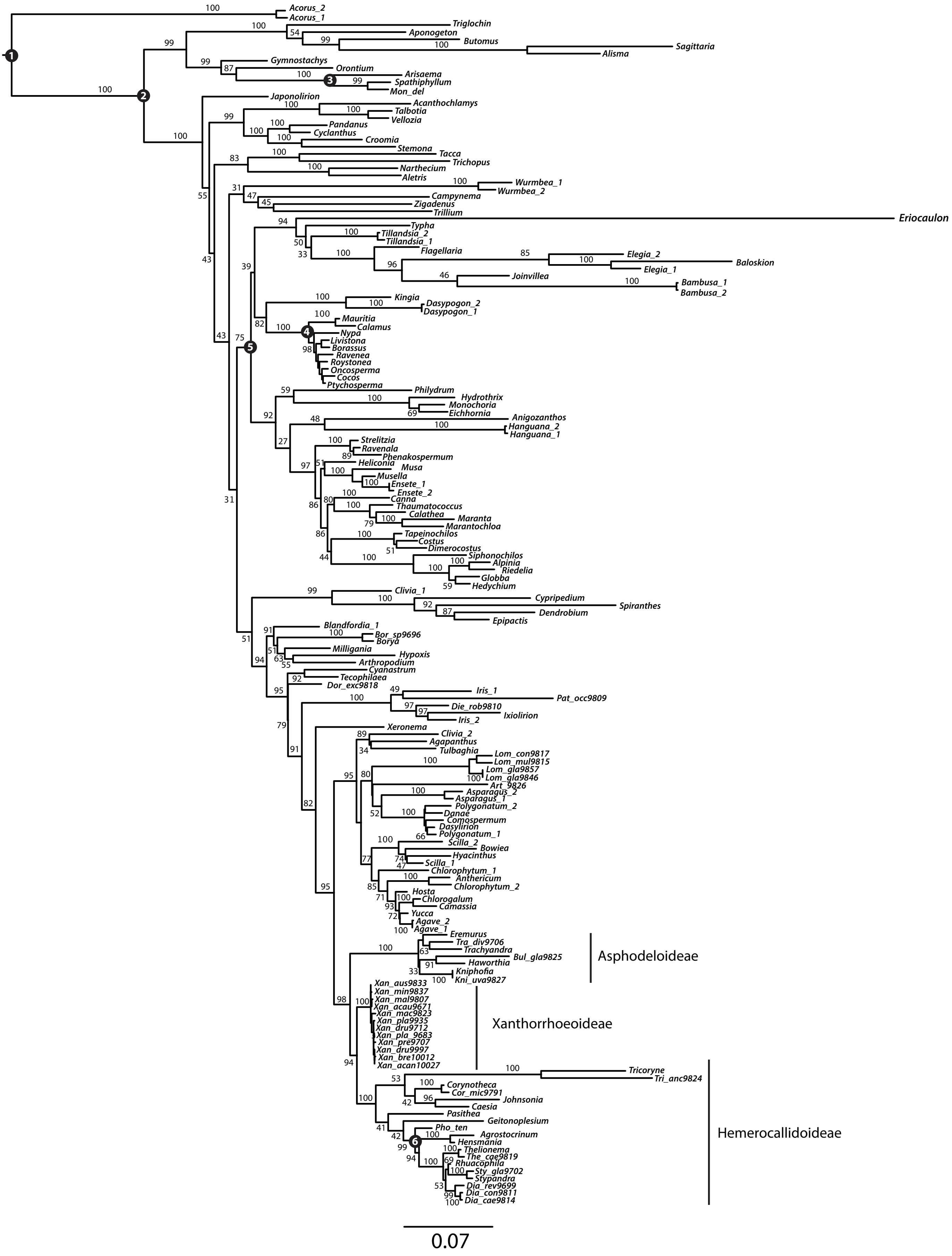

Figure S2. Maximum likelihood phylogeny (best tree) of monocots including Xanthorrhoeaceae (bottom of page, with subfamilies labelled). The tree was derived using RAxML from combined sequences of ndhF and trnL-trnF (cpDNA), showing branch lengths proportional to substitutions per site. The tree is rooted using Acorus, which is the sister group to the rest of monocots. Xanthorrhoeaceae is at bottom of tree, with subfamilies labelled. Branch lengths are proportional to substitutions per site. Labels on branches indicate bootstrap support (some values below 50 have been omitted to minimise clutter near tips). Circled numbers indicate calibration points explained in the text.

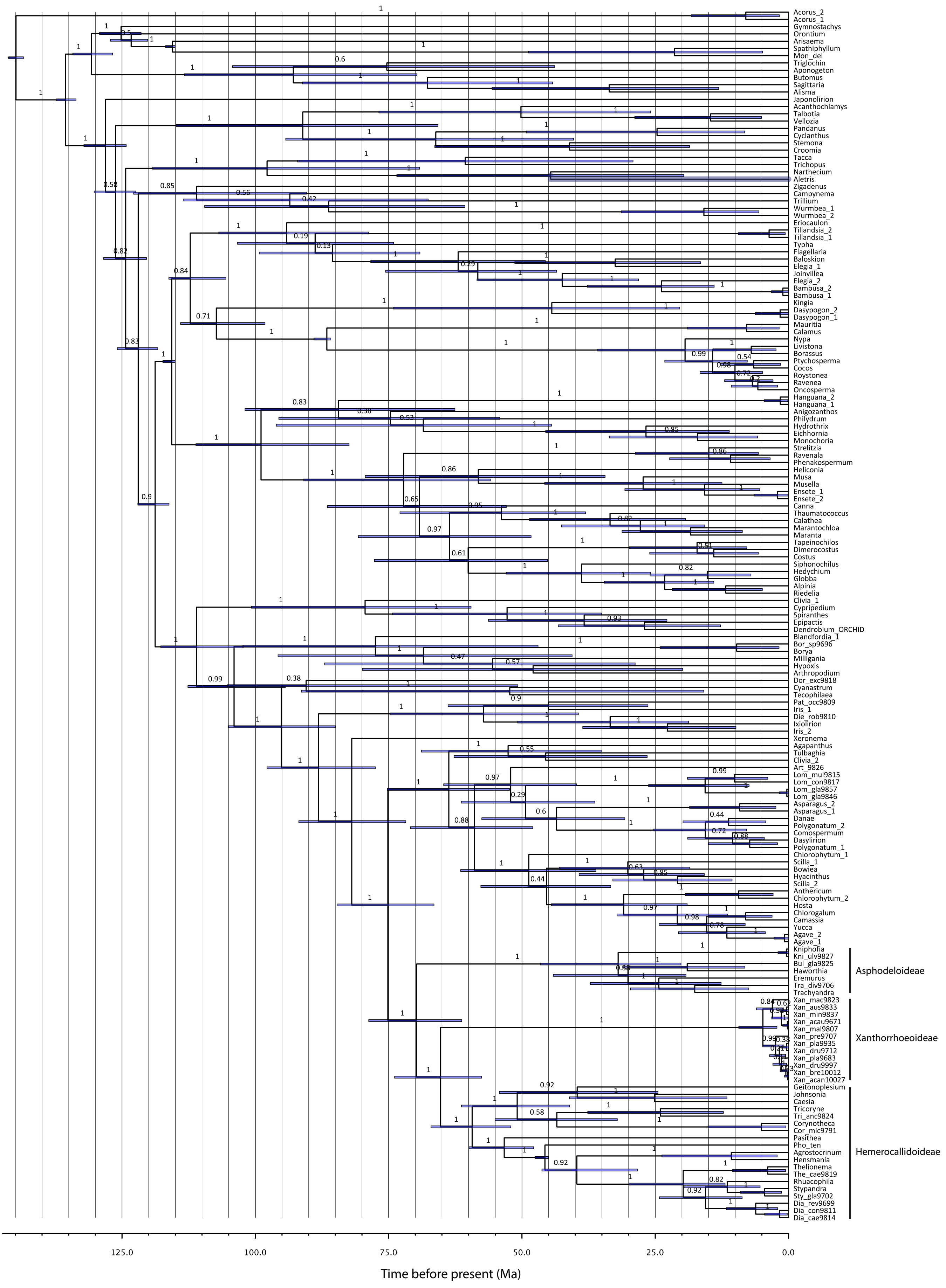

Figure S3. Uncorrelated lognormal clock (UCLN) chronogram of monocots including Xanthorrhoeaceae (bottom of page, subfamilies labelled) and derived from combined partitioned *ndhF* and trnL-trnF (chloroplast DNA) sequences using BEAST with a Yule tree model. Numbers on branches are are posterior probabilities and blue node bars are 95% BCIs of node height posterior estimates. The scale indicates time before present (Ma).

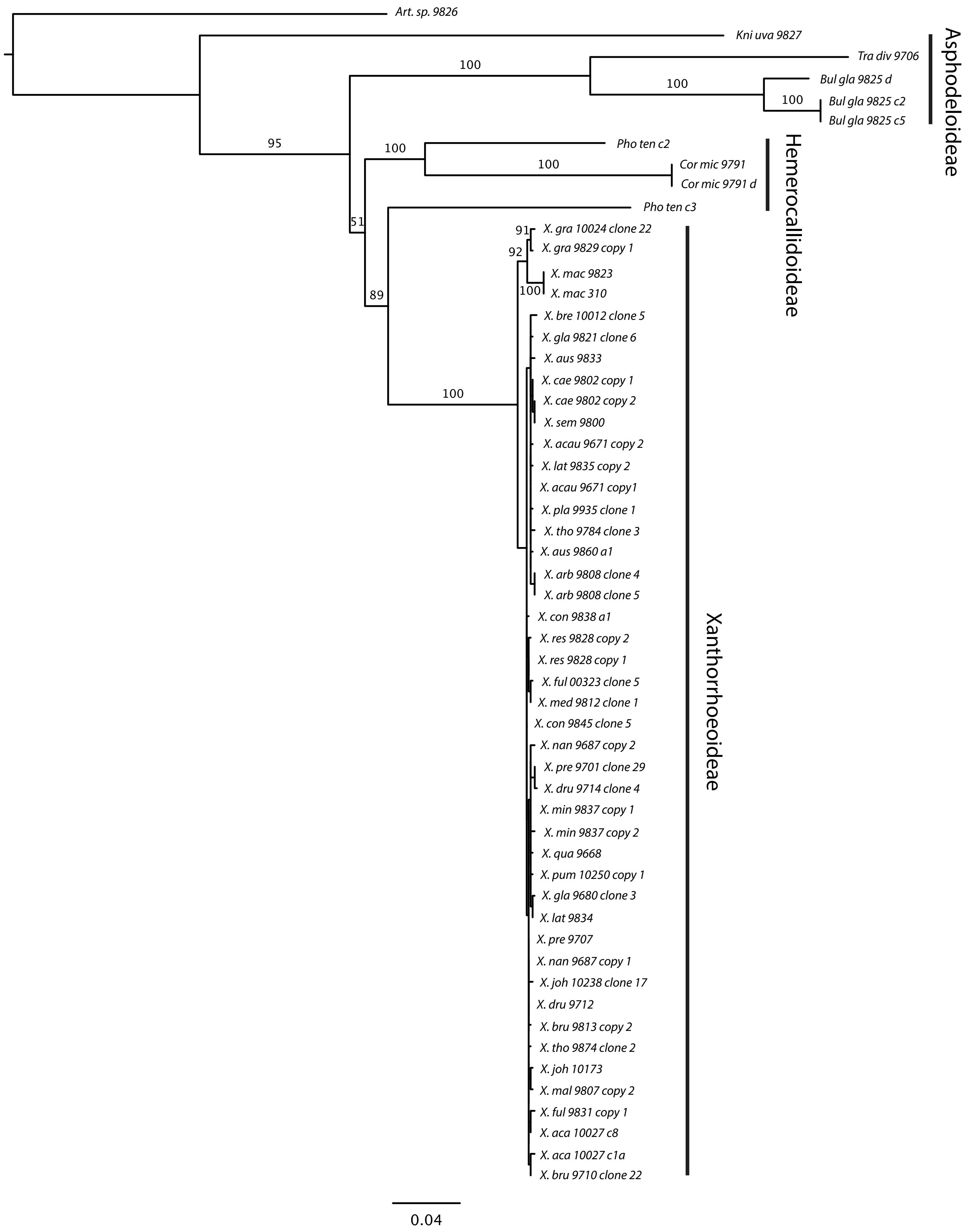

Figure S4. Maximum likelihood phylogeny of Xanthorrhoeaceae, derived using RAxML from sequences of *rpb2* (nDNA), showing branch lengths proportional to substitutions per site. The tree is rooted using *Arthropodium* (*Art. sp. 9826*) from the sister group, Asparagaceae. Clade labels with bars indicate subfamilies. Labels on branches indicate bootstrap support (most values within Xanthorrhoea have been omitted to minimise clutter.

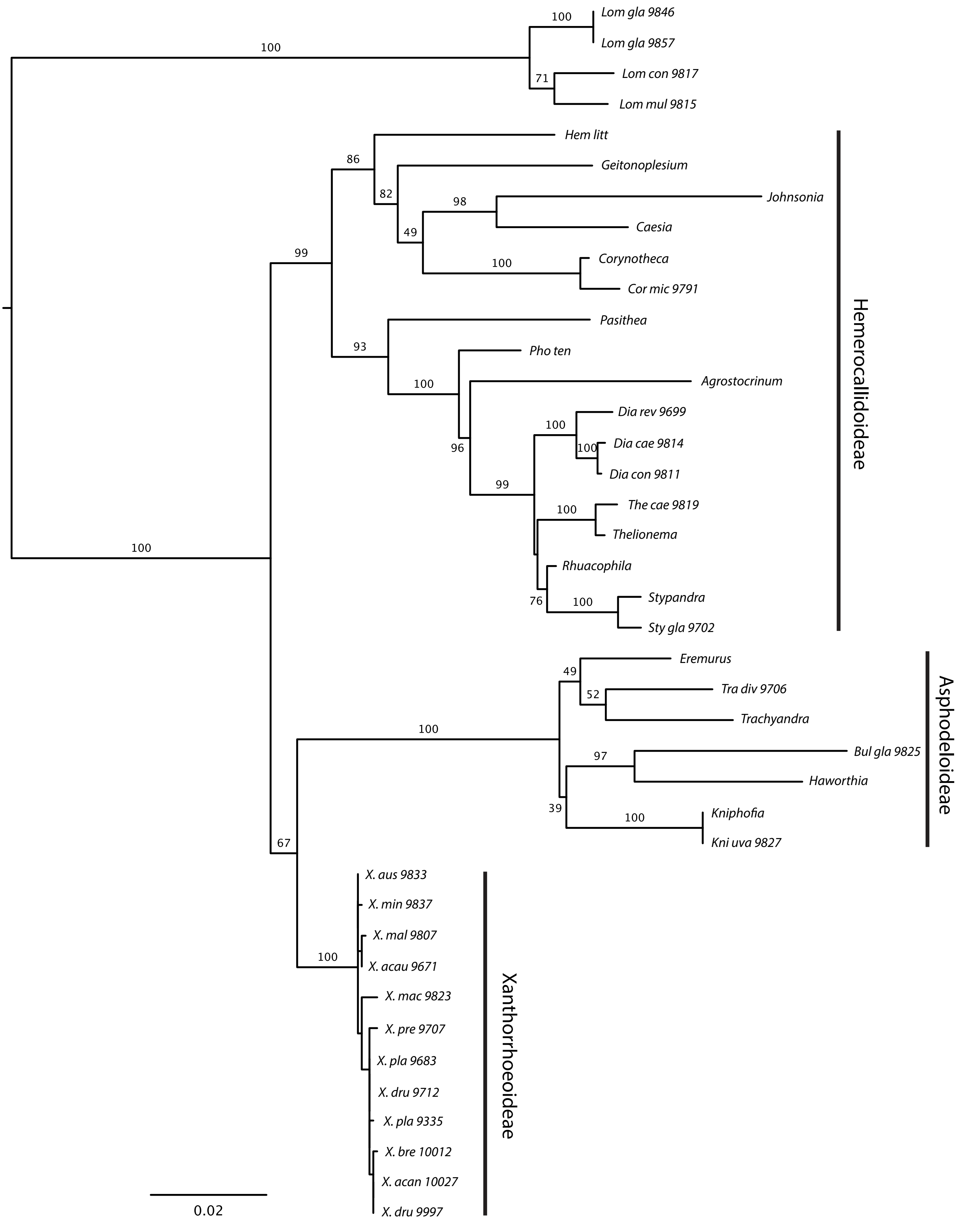

Figure S5. Maximum likelihood phylogeny of Xanthorrhoeaceae, derived using RAxML from combined partitioned *ndhF* and trnL-trnF (chloroplast DNA) sequences, showing branch lengths proportional to substitutions per site. The tree is rooted using *Lomandra* species from the sister group, Asparagaceae. Clade labels with bars indicate subfamilies. Labels on branches show bootstrap support (most values within *Xanthorrhoea* have been omitted to reduce clutter.

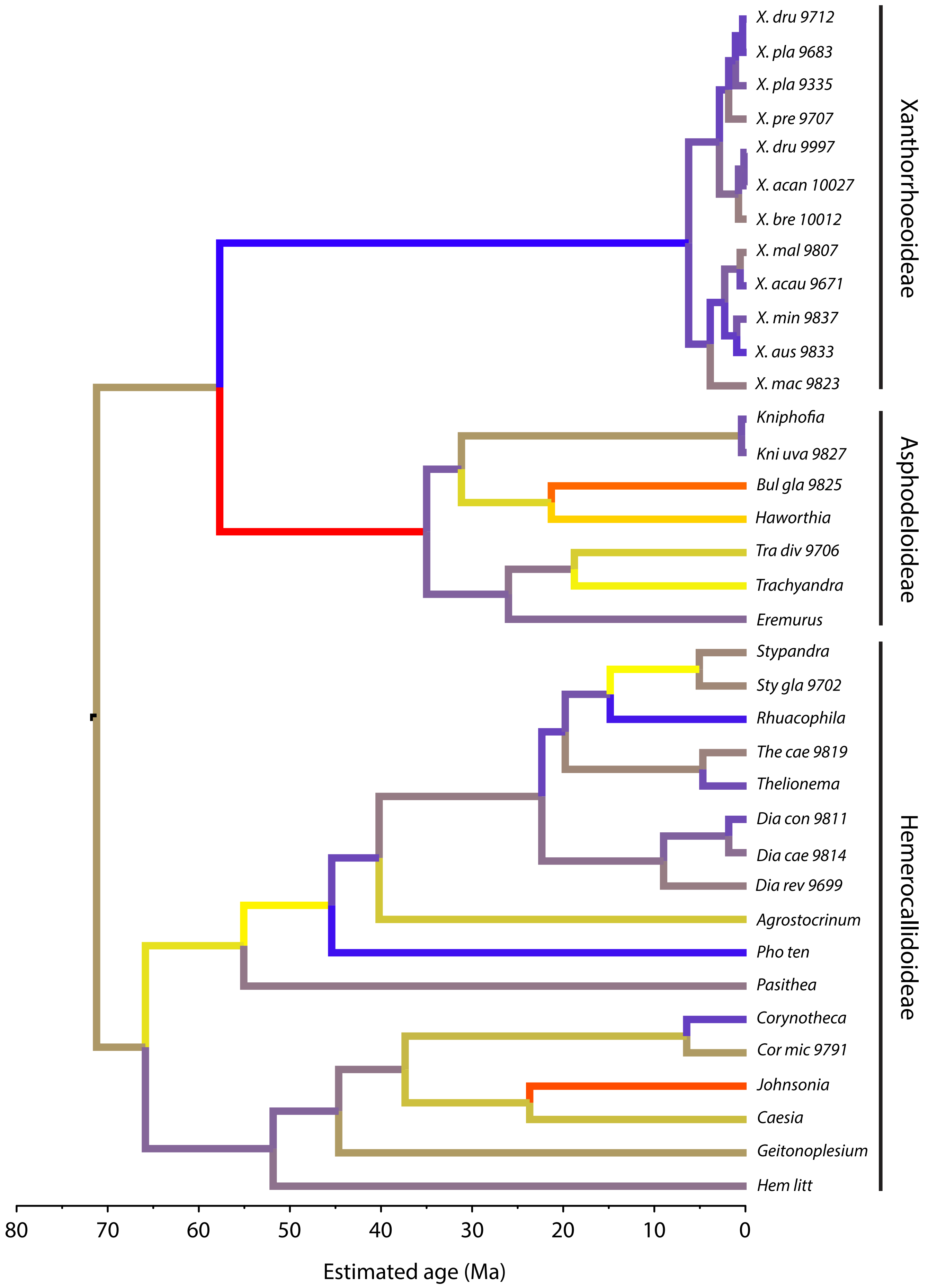

Figure S6. UCLN chronogram of Xanthorrhoeaceae derived from combined *ndhF* and trnL-trnF using BEAST with a Yule tree model. Branches are colored by median clock rates: red = fastest, blue = slowest, orange and green = intermediate. Clade labels with bars indicate subfamilies. The scale indicates time before present (Ma).

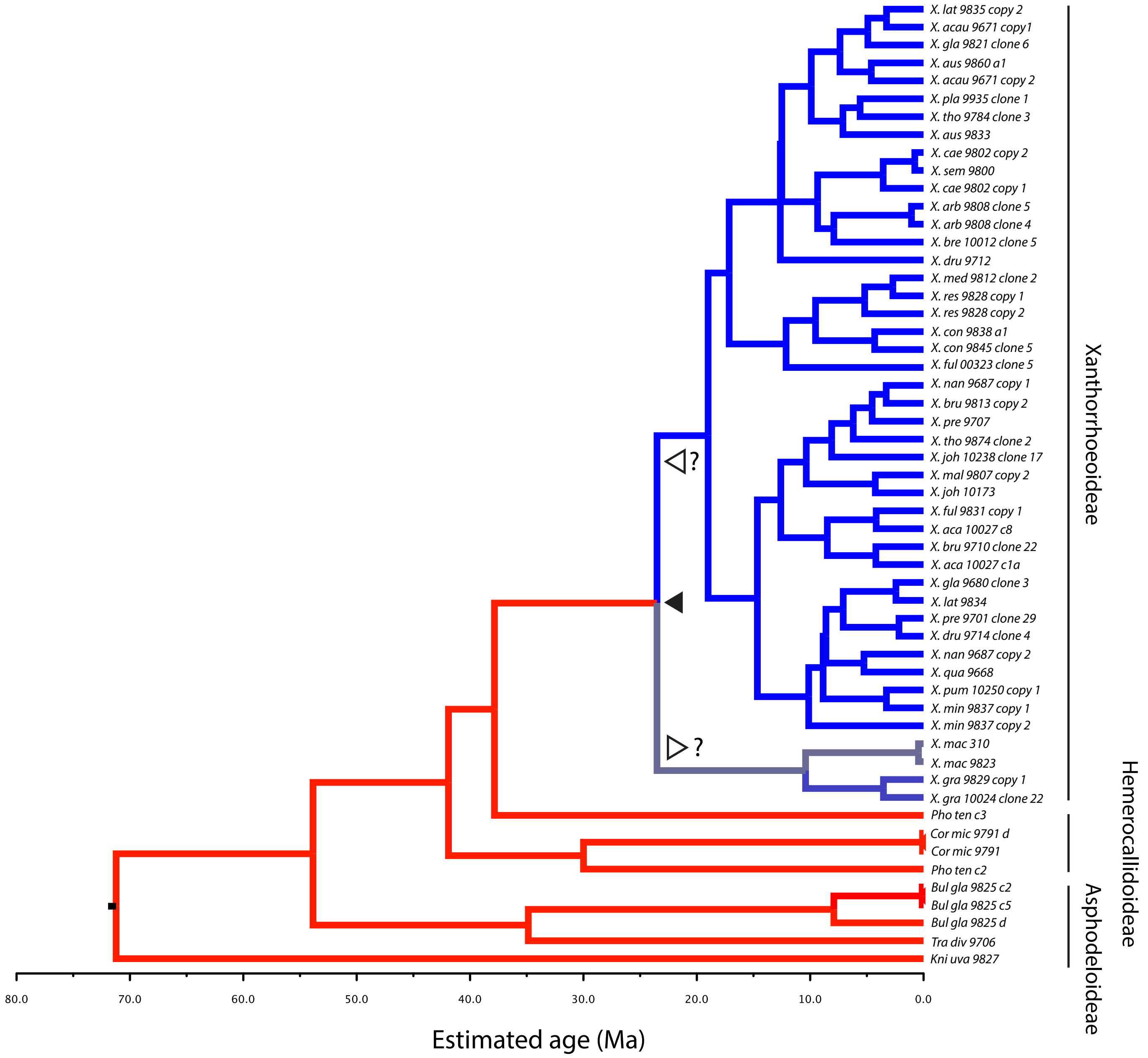

Figure S7. RLC chronogram of Xanthorrhoeaceae derived from sequences of *rpb2* (nuclear DNA) using BEAST with Yule tree model. Branches are colored by inferred local clock rates in the exons partition: red = fast, blue = slow. Pointers show the two inferred rate shifts: one downwards in the MRCA of Xanthorrhoeoideae (filled pointer), followed by a second shift (open pointers), which was either a further downward shift in the stem of the large upper clade or a small upward shift in the stem of the *X. macronema* + *X. gracilis* clade. Clade labels with bars indicate subfamilies. The scale indicates time before present (Ma).

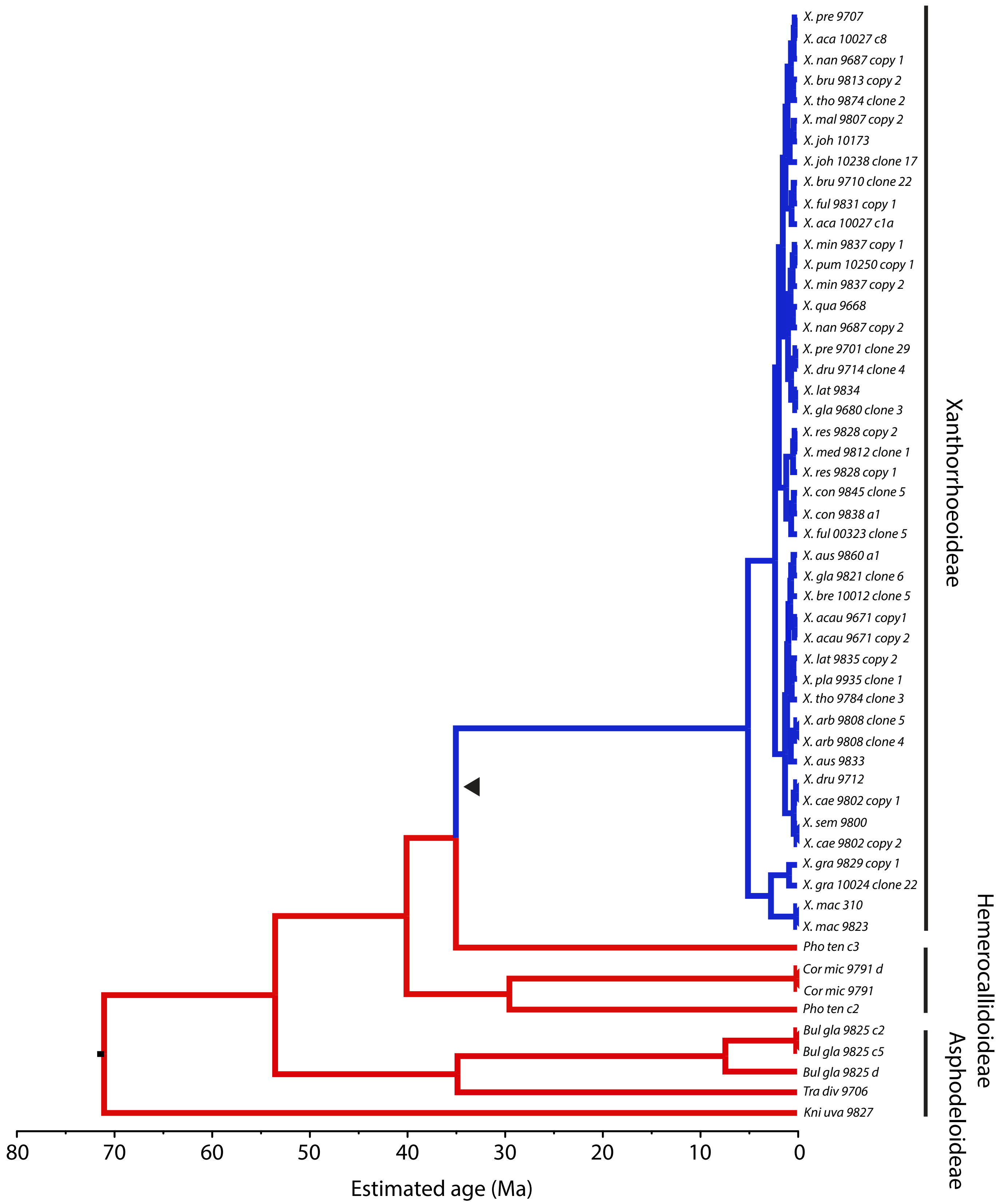

Figure S8. RLC chronogram of Xanthorrhoeaceae derived from sequences of *rpb2* (nDNA) using BEAST with a Birth-death tree model. Unlike other analyses of either dataset using the RLC clock, this one inferred a young age for the *Xanthorrhoea* crown (5.0 Ma). However, the model combination including a Yule clock was very strongly preferred for this dataset in Bayes factor tests. Branches are colored by inferred local clock rates in the introns partition: red = fast, blue = slow. The pointer shows the single inferred rate shift (downwards), which occurred in the MRCA of *Xanthorrhoea*. Clade labels with bars indicate subfamilies. The scale indicates time before present (Ma).

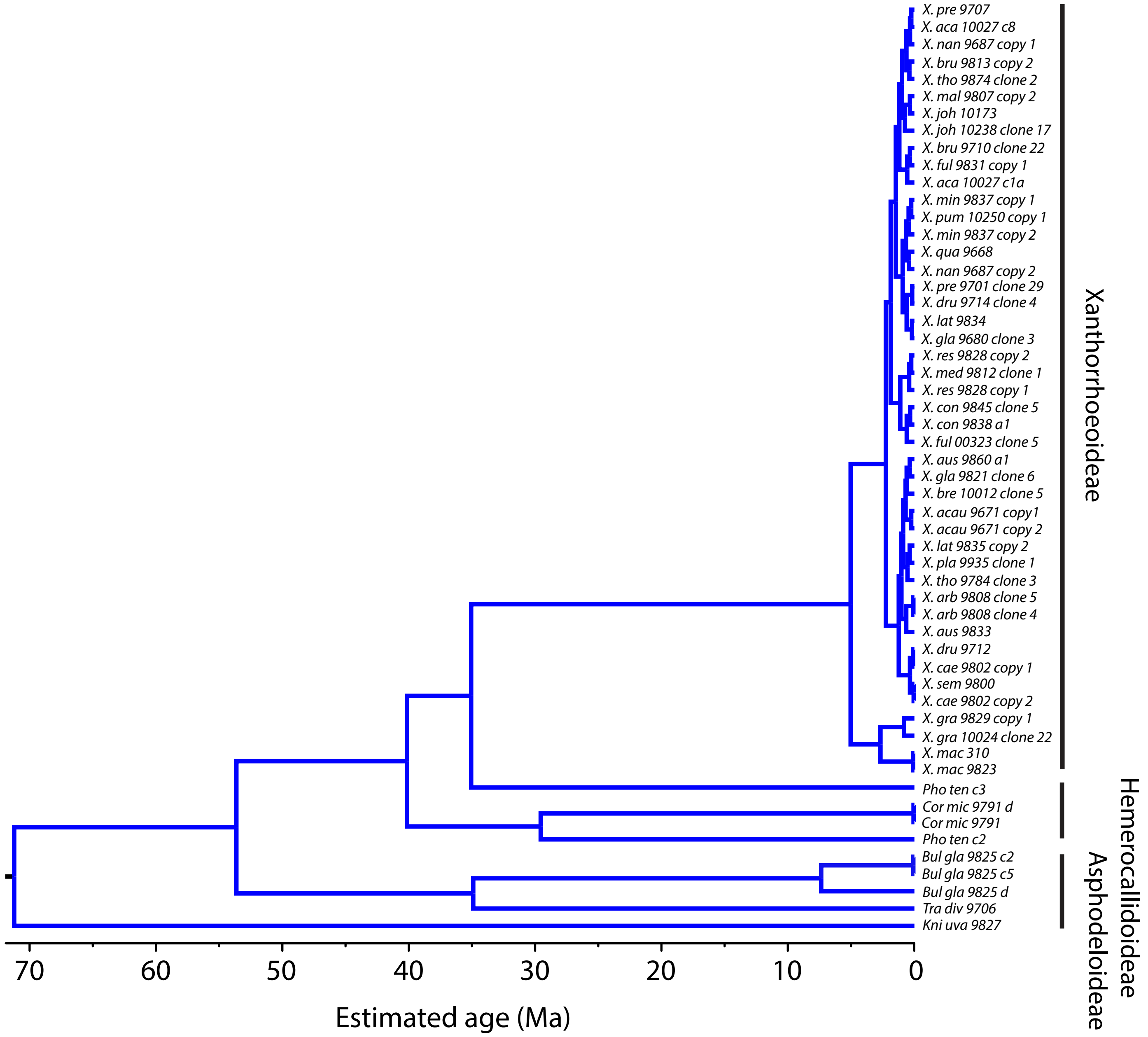

Figure S9. RLC chronogram of Xanthorrhoeaceae derived from sequences of *rpb2* (nDNA) using BEAST with a Birth-death tree model. Unlike other analyses of either dataset using the RLC clock, this one inferred a young age for the *Xanthorrhoea* crown (5.0 Ma). However, the model combination including a Yule clock was very strongly preferred for this dataset in Bayes factor tests. Branches are colored by inferred local clock rates in the exons partition: red = fast, blue = slow, i.e. in the exons, there is essentially no rate variation and the rate is much slower than in the introns. Clade labels with bars indicate subfamilies. The scale indicates time before present (Ma).

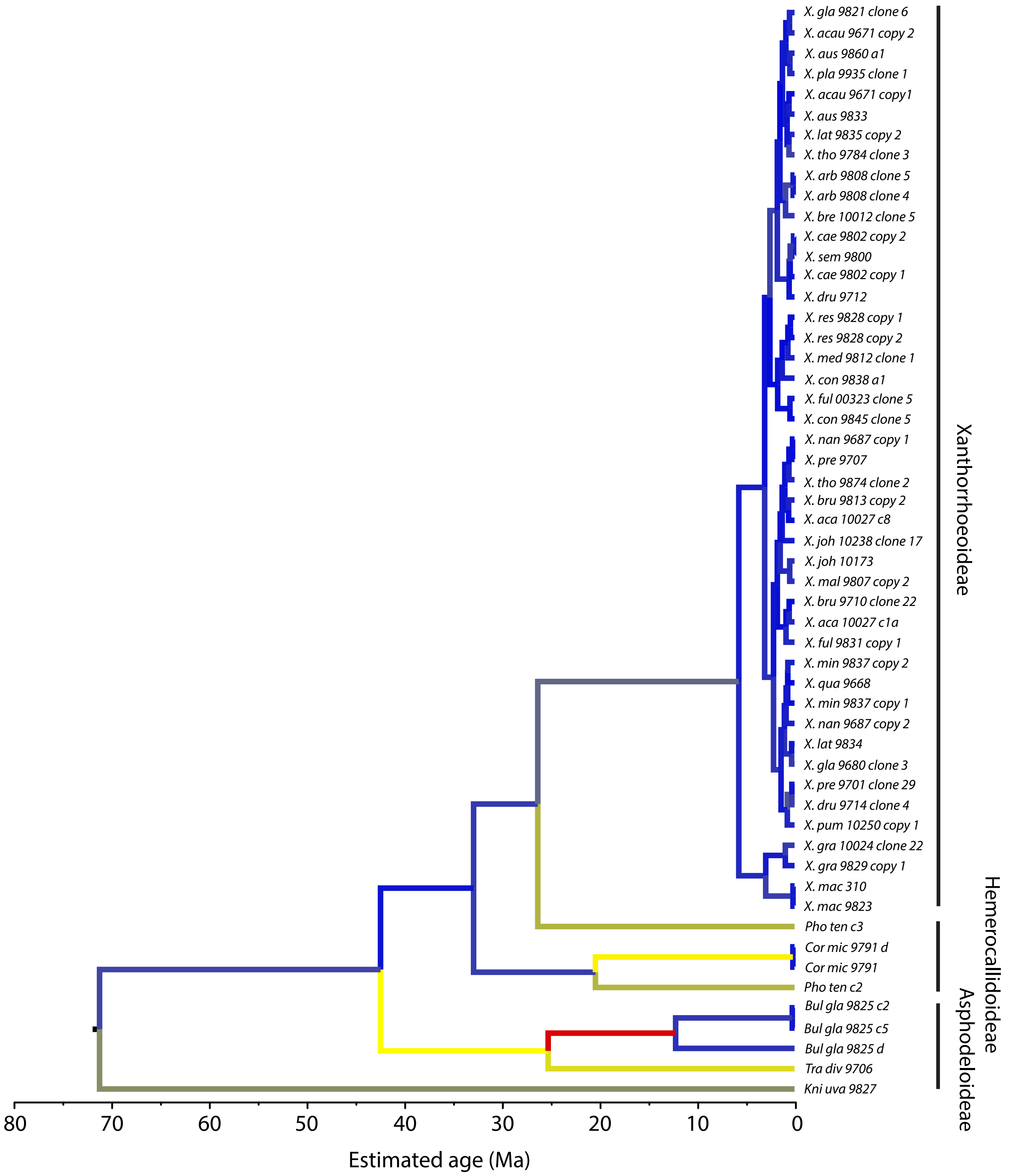

Figure S10. UCLN chronogram of Xanthorrhoeaceae derived from sequences of *rpb2* (nDNA) using BEAST with a Yule tree model. Branches are colored by median clock rates in the introns partition: red = fastest, blue = slowest, orange and green = intermediate. Clade labels with bars indicate subfamilies. The scale indicates time before present (Ma).

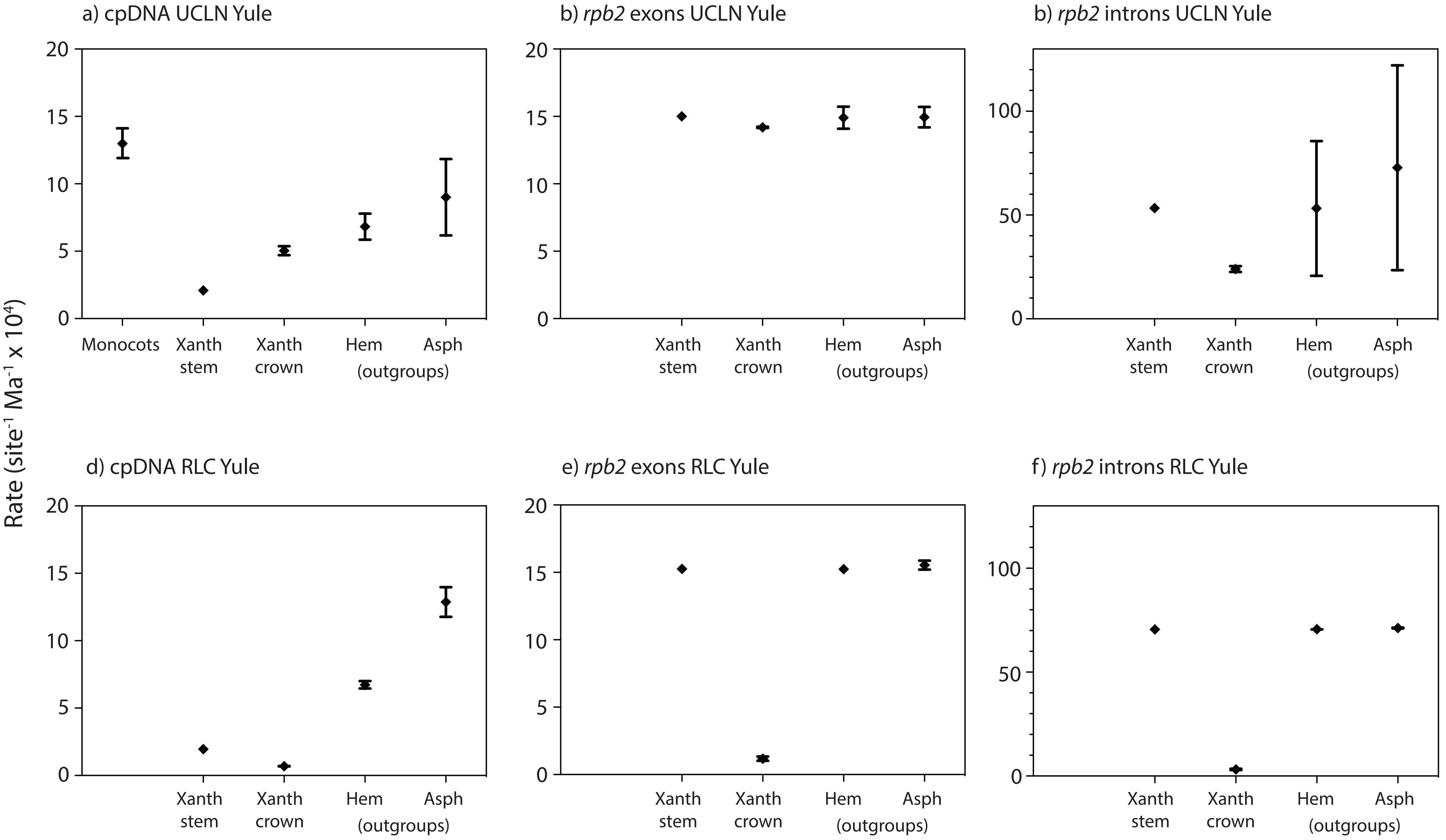

Figure S11. Estimates of DNA substitution rates in monocots, *Xanthorrhoea* and its two sister groups using BEAST for all three datasets (cpDNA-monocots, cpDNA-Xanthorrhoeaceae and *rpb2*-Xanthorrhoeaceae) under both alternative clock models (UCLN and RLC) and the Yule tree-growth model. For comparability of rates between the large (monocot) and small (Xanthorrhoeaceae) cpDNA datasets, neither was partitioned. It was not possible to estimate a rate using the RLC clock with the monocots-cpDNA dataset because none of the six analyses converged. Symbols represent means, and error bars the 95% CIs, of the "rate median" parameter values across all branches within the given taxon, as plotted on the annotated maximum clade credibility tree using FigTree v1.4.0 (Rambaut, 2012). This could not be done for the *Xanthorrhoea* stem, which is a single node. Taxon abbreviations: Xanth = Xanthorrhoea; Hem = Hemerocallidoideae; Asph = Asphodeloideae.

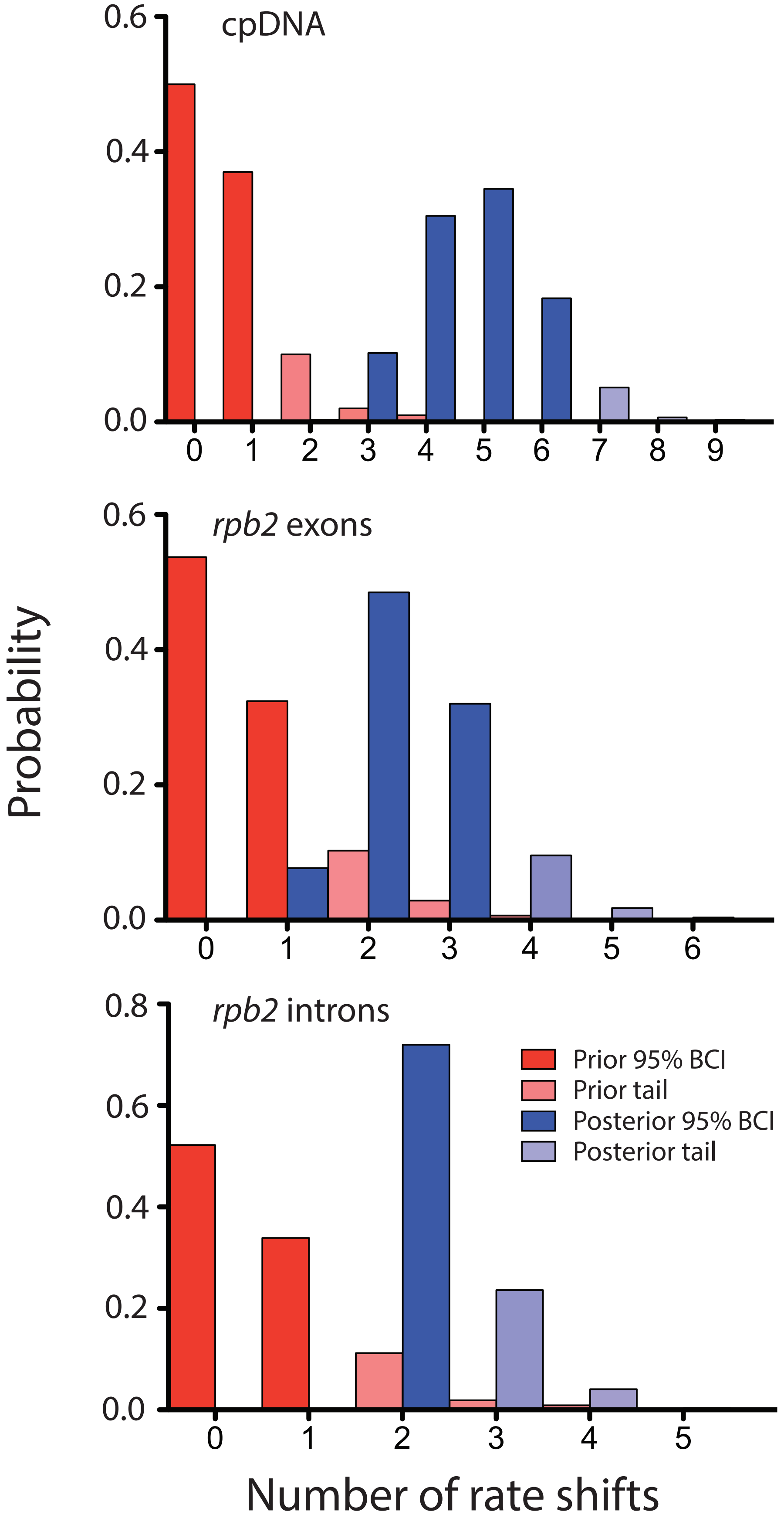

Figure S12. Prior and posterior distributions of number of rate shifts inferred by RLC clock analyses using BEAST from cpDNA and rpb2 sequences. The lack of overlap by the 95% Bayesian credibility intervals (BCIs) indicates rejection of the prior hypothesis of no rate shifts under a strict clock. Additionally, Fisher exact tests indicate that each pair of prior and posterior distributions is significantly different ($P = 0.0000$).
